# Associations of the circulating levels of cytokines with risk of ankylosing spondylitis: a Mendelian randomization study

**DOI:** 10.3389/fimmu.2023.1291206

**Published:** 2023-12-20

**Authors:** Yang Ye, Chuan-en Wang, Rui Zhong, Xiao-ming Xiong

**Affiliations:** Department of Spinal Surgery, Affiliated Sports Hospital of Chengdu Sport University, Chengdu, Sichuan, China

**Keywords:** cytokines, ankylosing spondylitis, Mendelian randomization, causal effect, CTACK, FGF-basic, G-CSF, MCP-3

## Abstract

**Background:**

Observational studies have shown that changes in circulating cytokine/growth factor levels occur throughout the initiation and progression of ankylosing spondylitis (AS), yet whether they are etiologic or downstream effects remains unclear. In this study, we performed a summarized-level bidirectional Mendelian randomization (MR) analysis to shed light on the causal relationship between the two.

**Methods:**

Genetic instrumental-variables (IVs) associated with circulating cytokine/growth factor levels were derived from a genome-wide association study (GWAS) of 8,293 European individuals, whereas summary data for the AS were obtained from a FinnGen GWAS of 166,144 participants. We used the inverse-variance-weighted (IVW) method as the main analysis for causal inference. Furthermore, several sensitivity analyses (MR-Egger, weighted median, MR-PRESSO and Cochran’s Q test) were utilized to examine the robustness of the results. Finally, reverse MR analysis was performed to assess reverse causality between AS and circulating cytokine/growth factor levels.

**Results:**

After Bonferroni correction, circulating levels of Cutaneous T-cell attracting (CTACK) and Monocyte specific chemokine 3 (MCP-3) were positively associated with a higher risk of AS (odds ratio [OR]: 1.224, 95% confidence interval [95% Cl]: 1.022 ~ 1.468, *P* = 0.028; OR: 1.250, 95% Cl: 1.016 ~ 1.539, *P* = 0.035). In addition, elevated circulating levels of Basic fibroblast growth factor (FGF-basic), Granulocyte colony-stimulating factor (G-CSF) and MCP-3 was considered a consequence of AS disease (β = 0.023, *P* = 0.017; β = 0.017, *P* = 0.025; β = 0.053, *P* = 0.025). The results of the sensitivity analysis were generally consistent.

**Conclusion:**

The present study supplies genetic evidence for the relationship between circulating cytokine levels and AS. Targeted interventions of specific cytokines may help to reduce the risk of AS initiation and progression.

## 1 Introduction

Ankylosing spondylitis (AS) is a common, highly inherited autoimmune disease in which the spine and pelvis are the main sites of involvement, and it is a subtype of axial spondylitis (1). Epidemiologic studies indicate that the average global prevalence of AS is around 0.5% (2). In addition to the tremendous physical and financial burden that AS imposes on patients, it also imposes a huge social burden (3). Despite the fact that the specific etiology and pathophysiologic mechanisms of AS are still unclear, as an autoimmune disease, a large number of studies have confirmed the important role of inflammation in its development and progression (4). Cytokines and growth factors (hereinafter referred to as cytokines) modulate the inflammatory response (5, 6), and therefore may be targeted for the prevention of AS. In fact, several biological disease-modifying antirheumatic drugs (bDMARD), such as interleukin-17 inhibitors (IL-17i) and tumor necrosis factor inhibitors (TNFi) (7, 8), have been tested in phase III clinical trials and recommended for use in patients with AS. Recently, several studies have provided a current summary of the pathogenesis of AS (9, 10). In particularly, immune responses mediated by cytokines play an important role. The fly in the ointment is that these studies are essentially from observational studies, and the results may be distorted by unpredictable confounding factors or reverse causation, making it difficult to establish a clear causal relationship (11).

Mendelian randomization (MR) study is an instrumental variable analysis that is used to reveal the causal effects of various genetically predictable exposures on complex diseases. This approach has the advantage of minimizing bias and reverse causality caused by confounding factors (12). Therefore, the method can be utilized to make causal inferences. To our knowledge, there are no MR study has evaluated the causal relationship between cytokines and AS. Given that a genome-wide association studies (GWAS) data containing 41 cytokines is publicly available (5), we therefore extracted validated genetic variants from it and performed a bidirectional MR study to explore the correlation between cytokines and AS.

## 2 Methods

### 2.1 Study design

**Figure 1:**
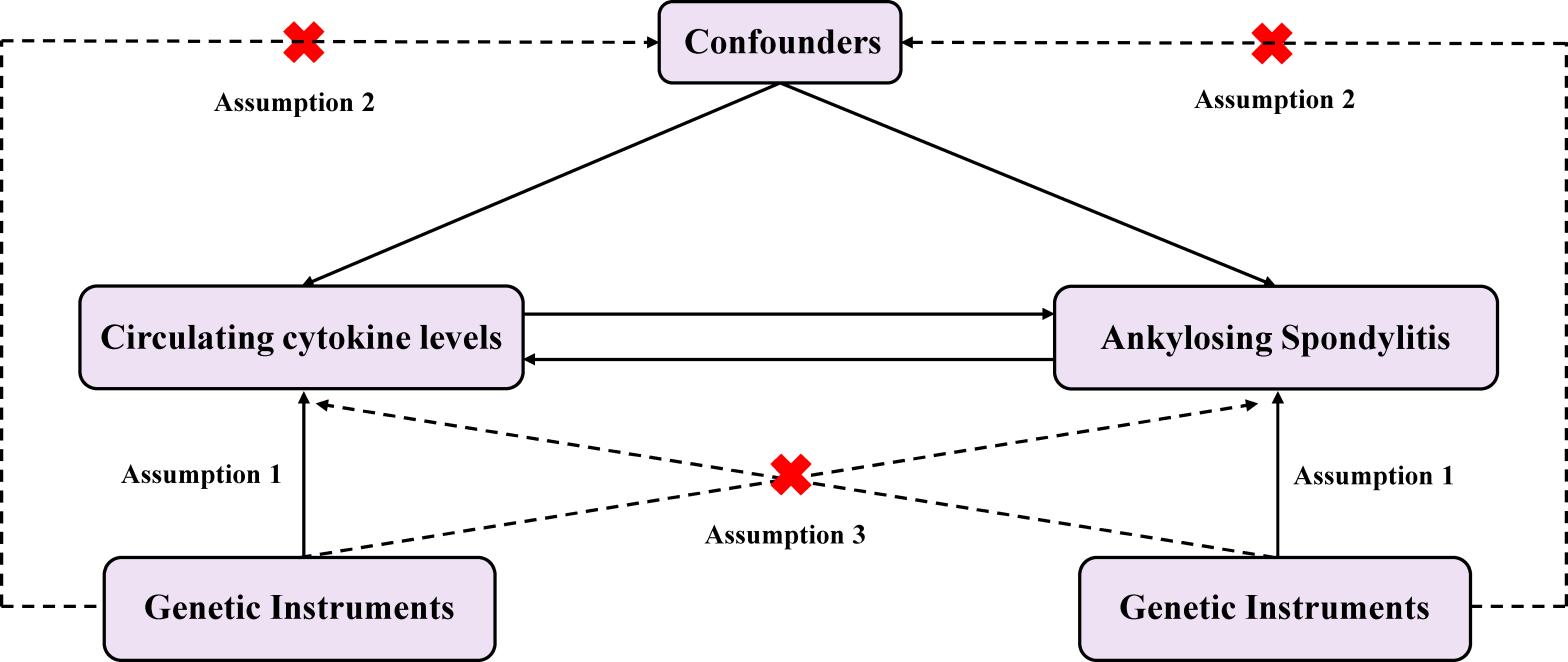
Diagram of the study design for bidirectional MR analysis. MR, mendelian randomization; SNPs, single nucleotide polymorphisms. Significant variables of 41 Circulating cytokine levels and Ankylosing spondylitis were selected to explore the bidirectional causal relationship. Assumption 1: The genetic variants (SNPs) are associated with exposure; Assumption 2: The genetic variants (SNPs) are independent of confounders; Assumption 3: The genetic variants (SNPs) affect outcome only by the exposure.

We conducted a two-sample MR design and single nucleotide polymorphisms (SNPs) as instrumental variables (IVs) for the cytokines. The summary-level data of GWAS on cytokines and AS were utilized to explain the causal relationship. Aiming to obtain unbiased causal effects, three key assumptions of this method include: 1) the SNPs are related to the exposure (cytokines); 2) the SNPs are independent of potential confounders; and 3) the SNPs affect the outcome (AS) only by the exposure (cytokines) (**Figure 1**).

### 2.2 Ethics

We used the publicly available GWAS summary-level data for the analysis, and each cohort enrolled in the GWAS study received ethical permission and consent to participate. Therefore, our study was exempt from review and approval by the research ethics committee.

### 2.3 Data sources and data preparation

#### 2.3.1 Cytokines

Summary statistics and IVs for circulating cytokine levels were obtained from a GWAS including 8293 Europeans (5). Participants were drawn from three independent population-based cohort studies (the Young Finns Cardiovascular Risk Study (YFS), FINRISK1997, and FINRISK2002) and were randomly selected from five different geographic regions in Finland. Besides, the researchers developed an additional genetic model, an additive model corrected for age, sex, body mass index, and the top ten ranked genetic principal components, to estimate the genetic association between 10.7 million SNPs and 41 circulating cytokine concentrations. Relevant details are presented in the **Supplementary material**-**Supplementary Table 1**. First, we selected all SNPs that independently and strongly (r²< 0.001, distance = 250 kb) predicted cytokines in a genome-wide sense (*P* < 5 × 10^-8^). Since the number of SNPs was too limited (n < 3) at *P* < 5 × 10^-8^ for some cytokines, to try to avoid false positives, we gradually used a relaxed significance threshold (*P* < 5 × 10^-7/-6^) to select IVs until a sufficient number of SNPs could be captured (n ≥ 3). After aggregation (r2 = 0.001, distance = 250 kb). Eventually, a total of 290 SNPs associated with 41 cytokines were identified. After removing the 47 SNPs that were not available in the AS dataset, 243 SNPs associated with 41 cytokines were finally used as IVs for the preliminary study. Details of the genetic variation of the 243 SNPs are listed in the **Supplementary materials**-**Supplementary Table 2**.

#### 2.3.2 Ankylosing spondylitis

We obtained summarized data for AS containing 166,144 individuals (ncase = 1462, ncontrol = 164,682) from the FinnGen consortium by integrating the Epidemiology Group Open GWAS database (**Supplementary material**-**Supplementary Table 1**). The FinnGen study is a national GWAS meta-analysis of data from 9 biobanks in Finland. The GWAS overlap with cytokines was limited (<3%). Therefore, we consider the risk of bias due to sample overlap to be minimal (13). For more details about FinnGen, see https://finngen.gitbook.io/documentation/. Ultimately, 13 genome-wide significant (*P* < 5 × 10^-8^) SNPs were identified as AS-independent IVs (r² = 0.001, distance = 250 kb). Details of the genetic variation of the 13 SNPs are listed in the **Supplementary materials**-**Supplementary Table 3**. Using these SNPs, we performed inverse MR analysis to investigate the effect of AS genetic susceptibility on circulating cytokine levels.

### 2.4 Statistical analysis

The Bonferroni method was used to perform a multiple comparison correction to calculate the statistical significance of a *P* -value < 1.22 × 10^-3^ (0.05/41) according to the number of cytokines. *P* -values between 1.22 x 10^-3^ and 0.05 were considered suggestive evidence of a potential causal association between the two (14). We performed the statistical analyses in R (version 4.3.1) with TwoSampleMR package (version 0.5.6) and MR-PRESSO package.

We used 41 cytokines as exposure factors and extracted relevant information (e.g., β-values and SE-values) for each SNP. First, the genetic variance (R²) of each cytokine was estimated using the information in the raw data (15). In addition, we calculated the F-statistic to quantify the strength of the IVs.

Then, we utilized the inverse-variance-weighted (IVW) method as the primary analysis to evaluate the potential causal relationship between cytokines and AS (16). We also performed several sensitivity analyses. First, we conducted MR-Egger regression to evaluate for directional pleiotropy (17), the intercept close to zero would be regarded as there is no directional pleiotropy. Second, we performed weighted median analysis (18), which has greater robustness to individual genetic instruments with strongly outlying causal evaluations. Third, we employed the MR-Pleiotropy RESidual Sum and Outlier (MR-PRESSO) test to identify and correct the outliers (19). Fourth, the Cochran’s Q test was employed to estimate the heterogeneity through the evaluates derived from each SNP (20). Fifth, to reduce the bias from confounders, we checked for the SNPs by using GWAS catalog (www.ebi.ac.uk/gwas/) and Phenoscanner Database (version 2) (http://www.phenoscanner.medschl.cam.ac.uk/) (21, 22). We reassessed causal effect estimates after excluding SNPs that were not associated with the current cytokine or that were simultaneously associated with multiple cytokines. Ultimately, we used the same methodology of inverse MR analysis of AS as an exposure factor and 41 cytokines as outcome factors to further explore the direction of causality.

## 3 Results

For the 41 circulating cytokines initially included in this study, only 10 cytokines had 3 or more genetic variants when we first selected SNPs with *P* < 5 × 10^-8^ and clustered at linkage disequilibrium (LD) r2 = 0.001. Further relaxation of the threshold was followed by another MR analysis (*P* < 5 × 10^-7/-6^), with 6 and 25 of the cytokines reaching our settings, respectively. Finally, all 41 cytokines were obtained with sufficient amount of valid genetic variants. The statistic for all IVs used in this study was more than 10 (11 ~ 789), indicating that the IVs were sufficiently strong (23). Complete data was placed in the **Supplementary material**-**Supplementary Tables 4**, **5**.

Two cytokines (CTACK, MCP-3) had a potential causal relationship with AS after Bonferroni correction. Genetically predicted higher circulating CTACK levels were associated with an increased risk of AS, (odds ratio [OR] = 1.224, 95% confidence interval [CI]: 1.033 ~ 1.451, *P* = 0.020). Weighted median method (OR = 1.256,95% CI: 1.024 ~ 1.589, *P* = 0.030). MR-Egger regression did not show the presence of directed pleiotropy (intercept *P* -value = 0.467). MR-PRESSO test did not report anomalies SNP and Cochran’s Q test suggested no evidence of heterogeneity (Cochran’s Q = 7.412; *P* = 0.493). Further searching the Phenoscanner database for IVs, we found 1 SNP (rs2731674) simultaneously associated with 24 phenotypes, but not with circulating CTACK levels. Therefore, we reran the MR analysis after excluding this SNP, the results of the IVW method showed no change in the potential causal relationship between the two (IVW OR = 1.224; 95% CI: 1.022 ~ 1.468; *P* = 0.028).

**Figure 2:**
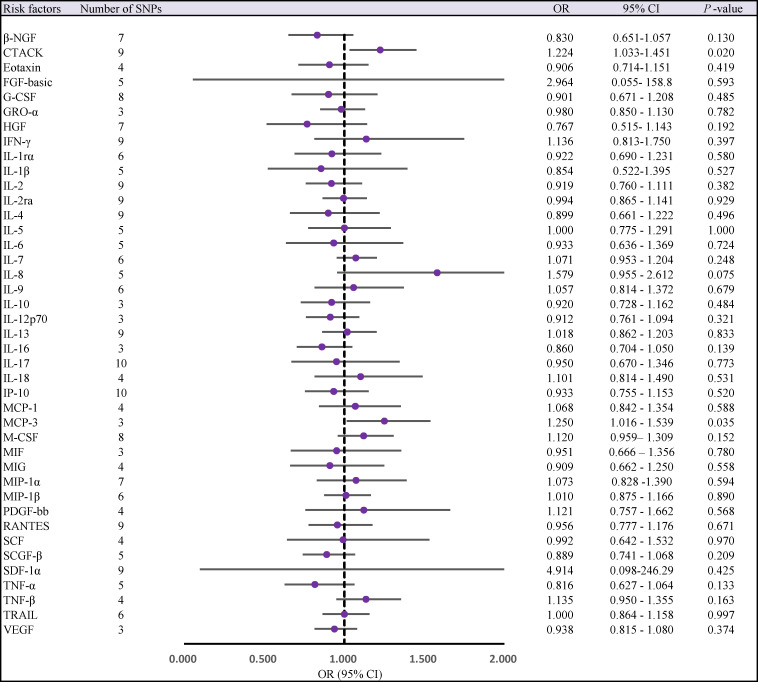
Forest plot of MR analysis of the relationship between 41 Circulating cytokine levels and risk of ankylosing spondylitis. CI, confidence interval; MR, mendelian randomization; OR, odds ratio; SNPs, single nucleotide polymorphisms.

**Figure 3:**
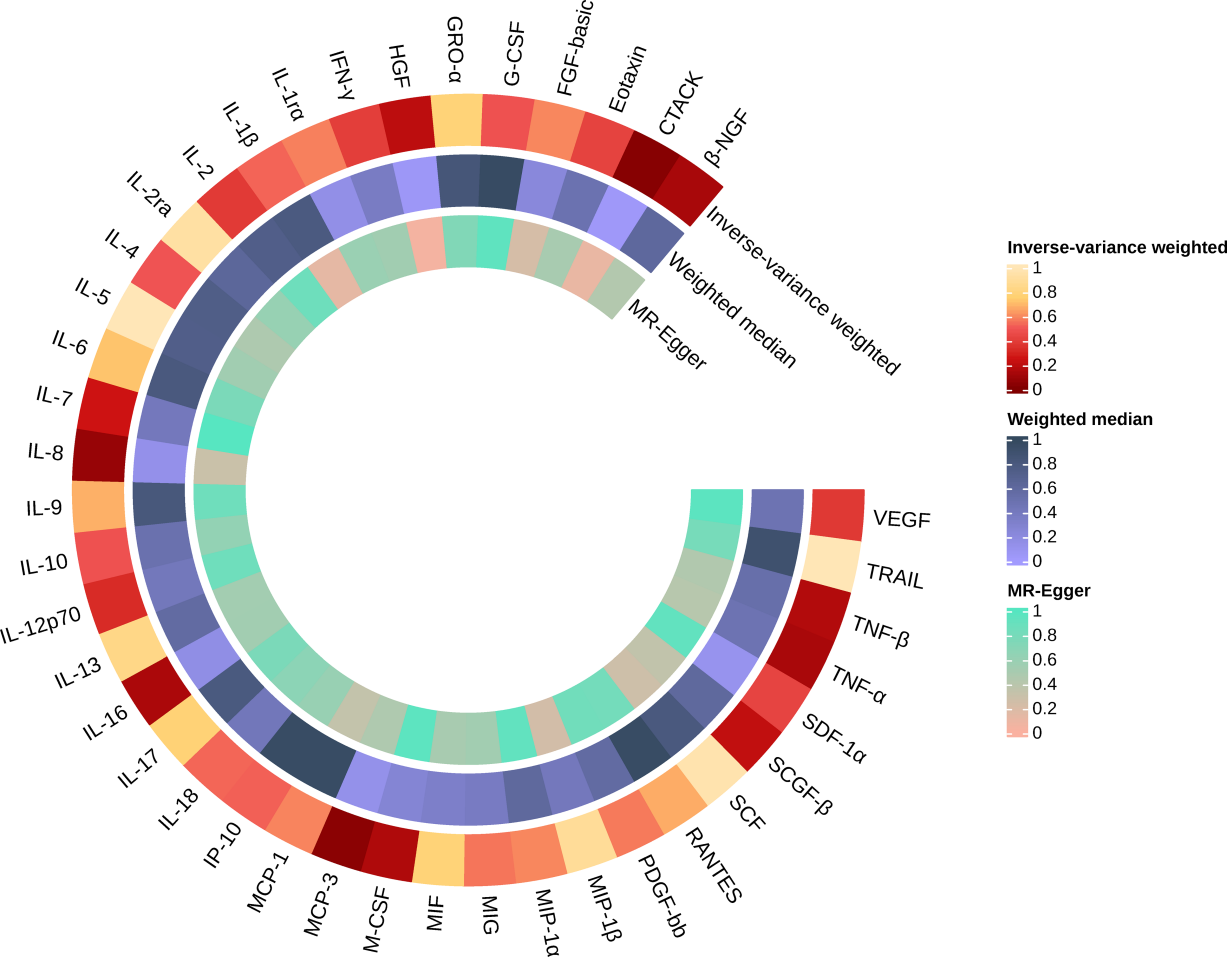
Causal effects of 41 Circulating cytokine levels on ankylosing spondylitis based on MR analysis. MR, Mendelian randomization. P values for variance inverse weighting, weighted median, and MR Egger are indicated from the outside in.

Similarly, we found that circulating MCP-3 levels were positively associated with the risk of AS by IVW method (OR = 1.250, 95% CI: 1.016 ~ 1.539, *P* = 0.035). Weighted median method (OR = 1.220, 95% CI: 0.948 ~ 1.571, *P* = 0.123). MR-Egger regression did not show the presence of directed pleiotropy (intercept P-value = 0.695). MR-PRESSO test did not report anomalies SNP and Cochran’s Q test suggested no evidence of heterogeneity (Cochran’s Q = 0.270; *P* = 0.874). On further searching for IVs in the Phenoscanner database, we found no pleiotropic SNPs. The results of the IVW methods reflecting the correlation of 41 cytokines with AS are presented separately in **Figures 2**, **3**. Moreover, the complete results of the MR analysis and other details are shown in the **Supplementary material**-**Supplementary Tables 5**–**8** and **Supplementary Figure 1**, **2**.

**Figure 4:**
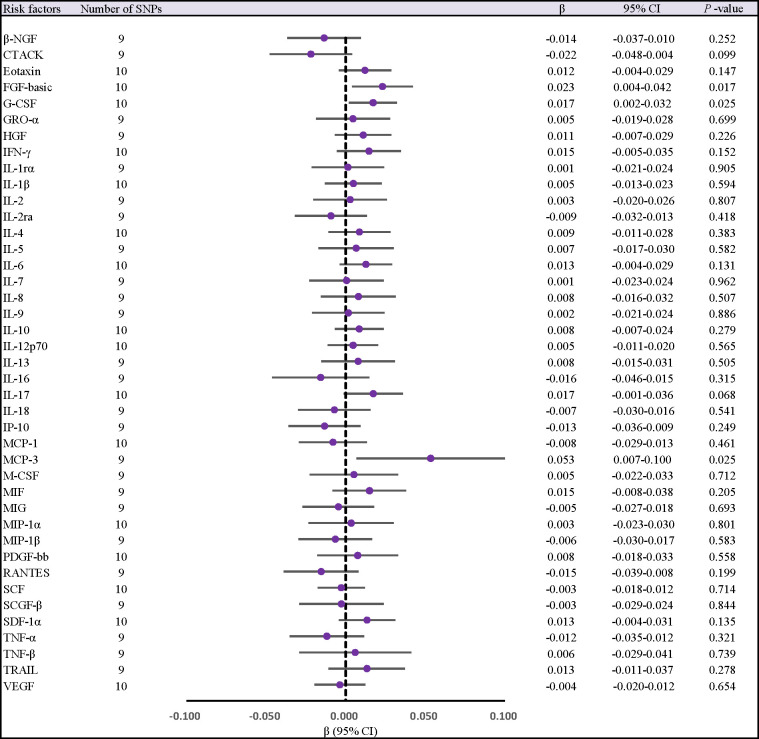
Forest plot of MR analysis of the relationship between ankylosing spondylitis and risk of 41 Circulating cytokine levels. CI, confidence interval; MR, mendelian randomization; OR, odds ratio; SNPs, single nucleotide polymorphisms.

**Figure 5:**
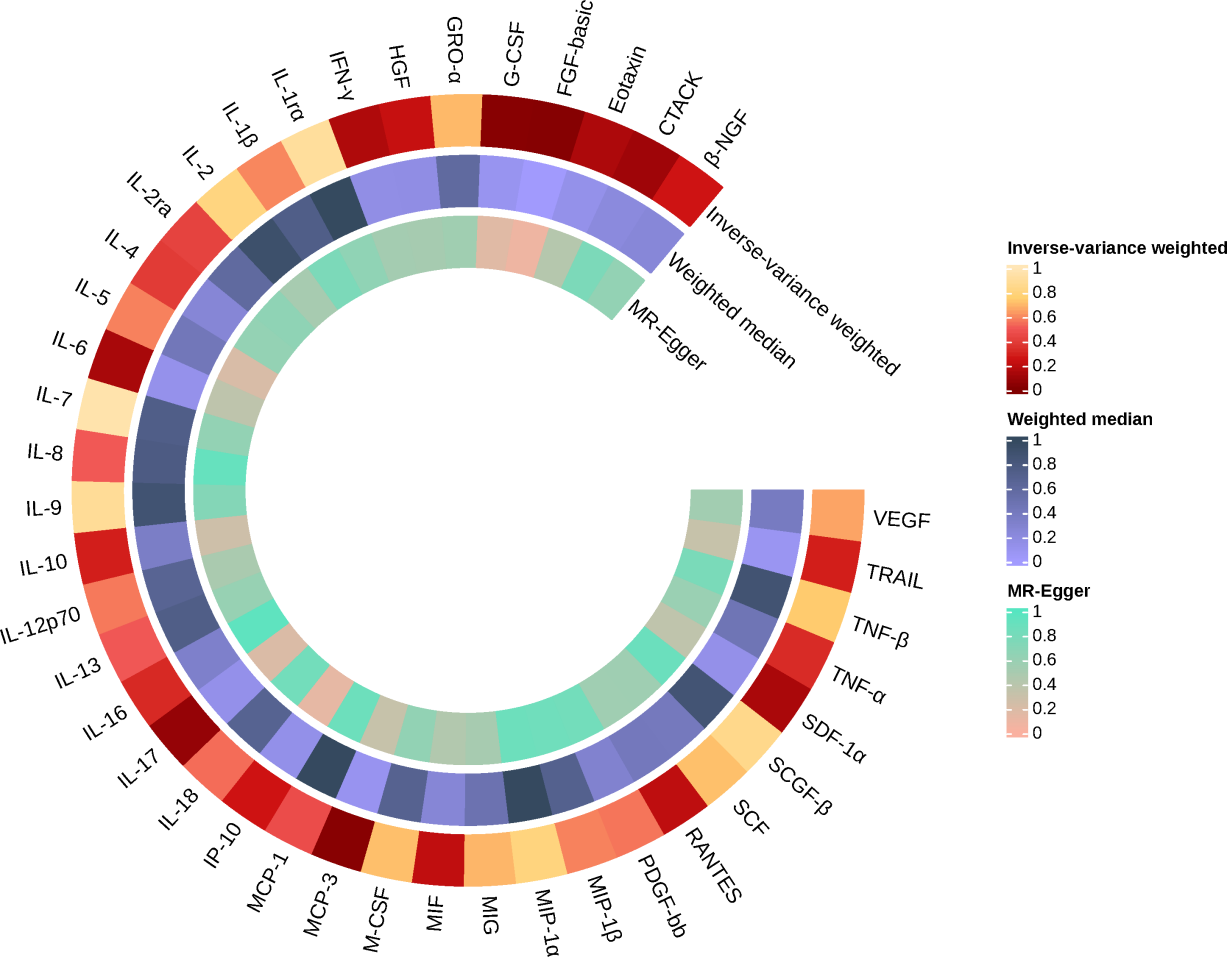
Causal effects of 41 Circulating cytokine levels on ankylosing spondylitis based on MR analysis. MR, Mendelian randomization. P values for variance inverse weighting, weighted median, and MR Egger are indicated from the outside in.

When 41 cytokines were used as outcome factors in the reverse MR, a total of 13 significant SNPs were retrieved as IVs for AS, all of which had F-statistics more than 10 (32 ~ 1408). But SNPs for incompatible alleles and palindromic SNPs for intermediate allele frequencies were filtered out in specific analyses. The results of the IVW method are presented in **Figures 4**, **5**. We observed a potential connection between AS and elevated circulating FGF-basic levels (IVW OR = 1.023, 95% CI: 1.004 ~ 1.043, *P* = 0.017), circulating G-CSF levels (IVW OR = 1.017, 95% CI: 1.002 ~ 1.033, *P* = 0.025), and circulating MCP-3 levels (IVW OR = 1.055, 95% CI: 1.007 ~ 1.105, *P* = 0.025), no evidence of directed pleiotropy, aberrant SNPs and heterogeneity was found. The results of the other methods can be found in the **Supplementary material**-**Supplementary Table 9** and **Supplementary Figures 3**–**5**.

## 4 Discussion

Our two-sample MR findings showed that genetically predicted higher levels of CTACK and MCP-3 were associated with higher risk of AS when cytokines were used as exposure factors and AS as an outcome factor. Whereas, genetically predicted AS was correlated with higher levels of FGF-basic, G-CSF and MCP-3 when AS was used as an exposure factor. There was a bidirectional causal relationship between higher MCP-3 levels and AS. Thus, the results of this study suggest that higher levels of CTACK may increase the incidence of AS, higher levels of FGF-basic and G-CSF may promote disease progression, and lifelong elevated MCP-3 levels have been implicated in both the onset of AS and disease progression.

CTACK, initially identified in keratinocytes in proliferative and inflammatory skin diseases (24). CTACK binds to its receptor CCR10 to recruit T cells to damaged skin and induce bone marrow-derived keratinocytes to participate in wound repair. In addition to skin diseases, CTACK plays a role in angiogenesis within tumors, proliferation and spread of tumor cells, and synergistically promotes lymphangiogenesis with VEGF (25, 26). In addition, observational studies have found elevated levels of CTACK in patients with idiopathic pulmonary fibrosis and suggested it as a predictor of disease prognosis (27). Studies on CTACK and autoimmune diseases are limited. Among them, observational studies have shown that restoration of the CTACK/CCR10 immunoregulatory impairments caused by psoriasis suppresses skin T-cell hyperactivation, thereby reducing the inflammatory response associated with psoriasis (28, 29). An *in vitro* study found that CTACK/CCL27 significantly inhibited the migration of human subchondral mesenchymal progenitor cells in synovial fluid to the site of microfracture defects, thereby adversely affecting the prognosis of RA patients (30). In addition, elevated CTACK/CCL27 concentrations have been found in patients with systemic sclerosis, neuromyelitis optica, and idiopathic inflammatory myopathies (31–33), and recent MR studies have similarly shown a positive correlation between CTACK/CCL27 concentrations and the prevalence of systemic lupus erythematosus (34), but studies are lacking to further elucidate these findings. Our MR similarly showed that higher CTACK levels were linked to a higher prevalence of AS; nevertheless, we found no previous reports of correlative studies between the two. Given the association of CTACK in other autoimmune diseases and the important role of other cytokines (e.g., IL-23, IL-7, and TNF-α) in AS (4), we believe that it is valuable to further explore the connection between CTACK levels and AS pathogenesis.

In the present study, genes predicted higher levels of FGF-basic and G-CSF in AS patients. Our results corroborate a previous case-control study (ncase = 80, ncontrol = 21), which found significantly higher serum levels of FGF-basic in AS patients than in healthy individuals (35). Studies have shown that FGF-basic plays diverse functions in the regulation of angiogenesis and osteogenesis (36), including multiple aspects of angiogenesis (from basement membrane degradation to remodeling) (37), induction of bone marrow mesenchymal stem cells and local bone progenitor cell proliferation (38, 39), and regulation of osteoblast-associated cell activity in concert with bone morphogenetic protein-2 (35). Pathological bone proliferation in the sacroiliac joints and spine are part of the progression of AS, in which the fibroblast growth factors may play an important role (4). Taken together, FGF-basic may be an attractive target for inhibiting AS progression.

We did not find evidence of a direct correlation between AS and G-CSF levels from previous studies. However, G-CSF levels have been observed to be significantly elevated in other autoimmune diseases. Nakamura et al.’s study found that synovial and serum levels of G-CSF were significantly higher in patients with rheumatoid arthritis than in normal subjects and indicated that this alteration was positively correlated with the activity of disease (40). Similarly, Damiati et al. observed the same results in the serum of female SLE patients (41). In addition, it has been found that elevated G-CSF levels enhance inflammatory and autoimmune responses in patients with vasculitis (42). G-CSF is produced by stromal cells in the bone marrow (BM) and plays a key regulatory role in neutrophil biology, from precursor production, differentiation and release in the BM to the function of mature neutrophils in different settings (43). Recent studies have found that upregulation of neutrophils may exacerbate the course of ankylosing spondylitis (44). Consequently, G-CSF may be a potential target in the inhibition of neutrophil-mediated AS disease progression. Furthermore, other cytokines (e.g., IL-17, IL-23, IL-6, TNF-α) upregulate the production and release of G-CSF (42, 45), and the levels of these cytokines have been shown to correlate with AS disease activity (4). Thus, G-CSF and other cytokines may interact with each other during the disease progression of AS, and further studies are warranted to elucidate the mechanism of their interaction.

Our MR findings show that MCP-3/CCL7 is the only cytokine with a bidirectional causal relationship with AS. Previously, only one case-control study was found to observe significantly elevated MCP-3/CCL7 levels in the serum of AS patients (46), but further studies between the two were lacking.MCP-3/CCL7 is a chemokine that attracts monocytes during inflammation and metastasis. MCP-3/CCL7 was found to be involved in the IL-17 signaling pathway, and overexpression of MCP-3/CCL7 exacerbates the progression of RA (47). Therefore, inhibition of the IL-17-CCL7 signaling pathway may be a promising therapeutic target for RA. In addition, animal experiments revealed that leukocyte migration in experimental autoimmune encephalomyelitis could be inhibited by targeting MCP-3/CCL7 thereby preventing disease progression (48). Our MR study found a potential causal relationship between AS and higher MCP-3/CCL7 levels, which led us to hypothesize that overexpression of MCP-3/CCL7 may exacerbate the progression of AS that is similar with RA. Meanwhile, the results of this study suggest that genetically predicted higher MCP-3/CCL7 levels are a risk factor for AS, and the possible explanation is that overexpressed MCP-3/CCL7 recruits excess immune cells to the sacroiliac joints or the peri-spinal region (49), which promotes the development of AS. It is evident that the role of MCP-3/CCL7 in AS may be underestimated, and our findings may provide some valuable references for subsequent larger-scale studies.

Our study has several advantages. First, we use a two-sample bidirectional MR approach to build causal inferences that minimizes confounders and reverse causation. Second, it revealed novel associations between CTACK, MCP-3, FGF-basic and G-CSF and AS risk. Deciphering the interactions between various cytokines and AS will enrich our understanding of the disease. Third, it suggests new directions for future research to validate and further explore these findings. However, some limitations are inevitable. First, when the significance threshold was set at (*P* < 5 x 10^-8^), we observed that higher HGF levels were associated with a reduced risk of AS (OR = 0.511, 95% CI: 0.322 ~ 0.811, *P* = 0.004), but the number of IVs was limited (SNPs = 2). In order to incorporate a larger number of instruments to improve statistical power, this relationship disappeared (OR = 0.767, 95% CI: 0.515 ~ 1.143, *P* = 0.192) when we chose a relaxed significance threshold (*P* < 5 x 10^-6^), and this may lead to an underestimation of the potential causal relationship between the two in our study. Second, the summarized data of AS used in the current study included a small number of patients with acute anterior uveitis with AS, which may have reduced the precision of the results. Third, although we used the GWAS catalog as well as the PhenoScanner to scan the IVs for potential secondary phenotypes, we could not completely exclude the possibility of pleiotropy. Fourth, the limited number of IVs may have resulted in a reduced ability to analyze MR sensitivity due to the overall small sample size for exposure and outcome factors. Fifth, our findings are only applicable to populations of European ancestry, and additional studies are needed for correlations in other ethnic groups. Therefore, the present this MR study is only a preliminary exploration of the causal relationship between cytokines and AS, and the results of the present study may be further validated or refuted when future relevant GWAS on a larger scale become available.

Finally, the present study has some clinical implications. Given the severe burden of AS on self and society, more research is still needed to find preventive strategies and more therapeutic strategies. Currently, favorable efficacy has been harvested by cytokine inhibitors (IL-17i, TNFi) to inhibit the development of AS (7, 8). Our study identified elevated concentrations of three cytokines associated with AS, and these findings may become new options for treating AS in the future. It is well known that prevention of AS is a common challenge facing the world. Our study found that elevated concentrations of 2 cytokines increase the risk of AS. It was reported that changes in lifestyle habits or certain diseases can cause changes in cytokine concentrations (33, 50). It is an attractive direction to investigate whether these changes lead to an increased risk of AS and whether cytokines play a mediating role. Thus, further clarification of the relationship between cytokines and AS may reveal ways to prevent the disease.

## 5 Conclusion

In the present study, the results suggest that genetically predicted higher circulating CTACK and MCP-3 levels are associated with an increased risk of AS. Meanwhile, higher circulating FGF-basic, G-CSF and MCP-3 levels were implicitly associated with AS development. Further studies, such as larger MR studies or clinical trials are necessary to validate these findings, elucidate underlying biological mechanisms and explore potential preventive and therapeutic targets.

## Data availability statement

The original contributions presented in the study are included in the article/**Supplementary Material**. Further inquiries can be directed to the corresponding author.

## Author contributions

YY: Data curation, Investigation, Methodology, Software, Writing – original draft. CW: Supervision, Writing – review & editing. RZ: Investigation, Writing – review & editing. XX: Conceptualization, Supervision, Writing – review & editing.
