# Supplementary material for: Associations of the circulating levels of cytokines with risk of ankylosing spondylitis: a Mendelian randomization study
Source: Front Immunol. 2023 Dec 20;14:1291206. doi: 10.3389/fimmu.2023.1291206 (PMC10761470; doi:10.3389/fimmu.2023.1291206)
Supplement: Supplementary file 1 [file DataSheet_1.docx]

Supplementary Materials

**Associations of the circulating levels of cytokines with risk of ankylosing spondylitis: a Mendelian randomization study**

**Yang Ye; Chuan-en Wang; Rui Zhong; Xiao-ming Xiong**

**Table S1.** Details of the genome-wide association studies and datasets used in this study. 2

**Table S2.** Details and *F*-statistics of genetic variants associated with cytokines. 5

**Table S3.** Characteristics of the genetic variants associated with the ankylosing spondylitis. 17

**Table S4.** Details of the number of genetic instruments, Variance explained and *F*-statistic for each cytokine. 18

**Table S5.** Characteristics of instrumental variables used for circulating levels of CTACK and MCP-3 in this study. 21

**Table S6.** Effect estimates of the associations between circulating levels of 41 cytokines and risk of ankylosing spondylitis in MR analyses. 22

**Table S7.** Details of the genetic variants with potential pleiotropy among instrumental variables of CTACK. 27

**Table S8.** Effect estimates for the association of circulating CTACK levels with risk of ankylosing spondylitis after exclusion of potential multidirectional SNPs. 29

**Table S9.** Effect estimates of the associations of ankylosing spondylitis with risk of circulating levels of FGF-basic, G-CSF and MCP-3. 30

**Figure S1.** MR Plots for Relationship of CTACK with AS. 31

**Figure S2.** MR Plots for Relationship of MCP-3 with AS. 32

**Figure S3.** MR Plots for Relationship of AS with FGF-basic. 33

**Figure S4.** MR Plots for Relationship of AS with G-CSF. 34

**Figure S5.** MR Plots for Relationship of AS with MCP-3. 35

**Table S1.** Details of the genome-wide association studies and datasets used in this study.

| Exposure or outcome | Abbreviations | | Sample size | Ancestry | | GWAS ID |  |
| --- | --- | --- | --- | --- | --- | --- | --- |
| Beta nerve growth factor | β-NGF | | 3,531 | European | | ebi-a-GCST004421 |  |
| Cutaneous T-cell attracting (CCL27) | CTACK | | 3,631 | European | | ebi-a-GCST004420 |  |
| Eotaxin (CCL11) | Eotaxin | | 8,153 | European | | ebi-a-GCST004460 |  |
| Basic fibroblast growth factor | FGF-basic | | 7,565 | European | | ebi-a-GCST004459 |  |
| Granulocyte colony-stimulating factor | G-CSF | | 7,904 | European | | ebi-a-GCST004458 |  |
| Growth regulated oncogene-α (CXCL1) | GRO-a | | 3,505 | European | | ebi-a-GCST004457 |  |
| Hepatocyte growth factor | HGF | | 8,292 | European | | ebi-a-GCST004449 |  |
| Interferon-gamma | IFN-γ | | 7,701 | European | | ebi-a-GCST004456 |  |
| Interleukin-1 receptor antagonist | IL-1rα | | 3,638 | European | | ebi-a-GCST004447 |  |
| Interleukin-1-beta | IL-1β | | 3,309 | European | | ebi-a-GCST004448 |  |
| Interleukin-2 | IL-2 | | 3,475 | European | | ebi-a-GCST004455 |  |
| Interleukin-2 receptor, alpha subunit | IL-2rα | | 3,677 | European | | ebi-a-GCST004454 |  |
| Interleukin-4 | IL-4 | | 8,124 | European | | ebi-a-GCST004453 |  |
| Interleukin-5 | IL-5 | | 3,364 | European | | ebi-a-GCST004452 |  |
| Interleukin-6 | IL-6 | | 8,189 | European | | ebi-a-GCST004446 |  |
| Interleukin-7 | IL-7 | | 3,409 | European | | ebi-a-GCST004451 |  |
| Interleukin-8 (CXCL8) | IL-8 | | 3,526 | European | | ebi-a-GCST004445 |  |
| Interleukin-9 | IL-9 | | 3,634 | European | | ebi-a-GCST004450 |  |
| Interleukin-10 | IL-10 | | 7,681 | European | | ebi-a-GCST004444 |  |
| Interleukin-12p70 | IL-12p70 | | 8,270 | European | | ebi-a-GCST004439 |  |
| Interleukin-13 | IL-13 | | 3,557 | European | | ebi-a-GCST004443 |  |
| Interleukin-16 | IL-16 | | 3,483 | European | | ebi-a-GCST004430 |  |
| Interleukin-17 | IL-17 | | 7,760 | European | | ebi-a-GCST004442 |  |
| Interleukin-18 | IL-18 | | 3,636 | European | | ebi-a-GCST004441 |  |
| Interferon gamma-induced protein 10 (CXCL10) | IP-10 | | 3,685 | European | | ebi-a-GCST004440 |  |
| Monocyte chemotactic protein-1 (CCL2) | MCP-1 | | 8,239 | European | | ebi-a-GCST004438 |  |
| Monocyte specific chemokine 3 (CCL7) | MCP-3 | | 843 | European | | ebi-a-GCST004437 |  |
| Macrophage colony-stimulating factor | M-CSF | | 840 | European | | ebi-a-GCST004436 |  |
| Macrophage migration inhibitory factor (glycosylation-inhibiting factor) | MIF | | 3,494 | European | | ebi-a-GCST004434 |  |
| Monokine induced by interferon-gamma (CXCL9) | MIG | | 3,685 | European | | ebi-a-GCST004435 |  |
| Macrophage inflammatory protein-1α (CCL3) | MIP-1α | | 3,552 | European | | ebi-a-GCST004433 |  |
| Macrophage inflammatory protein-1β (CCL4) | MIP-1b | | 8,243 | European | | ebi-a-GCST004423 |  |
| Platelet derived growth factor BB | PDGF-bb | | 8,293 | European | | ebi-a-GCST004432 |  |
| Regulated on activation, normal T Cell expressed and secreted (CCL5) | RANTES | | 3,421 | European | | ebi-a-GCST004431 |  |
| Stem cell factor | SCF | | 8,290 | European | | ebi-a-GCST004429 |  |
| Stem cell growth factor beta | SCGF-β | | 3,682 | European | | ebi-a-GCST004428 |  |
| Stromal cell-derived factor-1 alpha (CXCL12) | SDF-1α | | 5,998 | European | | ebi-a-GCST004427 |  |
| Tumor necrosis factor-alpha | TNF-α | | 3,435 | European | | ebi-a-GCST004426 |  |
| Tumor necrosis factor-beta | TNF-β | | 1,559 | European | | ebi-a-GCST004425 |  |
| TNF-related apoptosis inducing ligand | TRAIL | | 8,186 | European | | ebi-a-GCST004424 |  |
| Vascular endothelial growth factor | VEGF | | 7,118 | European | | ebi-a-GCST004422 |  |
| Ankylosing spondylitis | AS | 1,462 cases and 164,682 controls | | European | finn-b-M13_ANKYLOSPON | |  |

Abbreviations: GWAS, genome-wide association studies; ID, Identifier.

**Table S2.** Details and *F*-statistics of genetic variants associated with cytokines.

| Cytokines | SNP | Beta. exposure | Se. exposure | Beta. outcome | Se. outcome | *F*-statistic |
| --- | --- | --- | --- | --- | --- | --- |
| β-NGF | rs28637706 | -0.159 | 0.026 | 0.005 | 0.041 | 36.504 |
|  | rs67476890 | 0.177 | 0.038 | -0.129 | 0.060 | 21.786 |
|  | rs71641308 | 0.204 | 0.043 | 0.010 | 0.064 | 22.365 |
|  | rs72780728 | 0.188 | 0.040 | -0.045 | 0.064 | 21.832 |
|  | rs73472576 | 0.118 | 0.025 | -0.013 | 0.038 | 21.963 |
|  | rs7970581 | -0.138 | 0.028 | 0.078 | 0.042 | 23.947 |
|  | rs9436119 | -0.112 | 0.025 | -0.026 | 0.038 | 20.765 |
| CTACK | rs116303454 | 0.383 | 0.082 | 0.027 | 0.118 | 22.030 |
|  | rs145902143 | 0.284 | 0.058 | 0.037 | 0.097 | 23.860 |
|  | rs2070074 | -0.447 | 0.037 | -0.129 | 0.059 | 142.656 |
|  | rs2731674 | 0.133 | 0.027 | 0.028 | 0.042 | 24.925 |
|  | rs3766110 | 0.129 | 0.028 | 0.023 | 0.043 | 21.432 |
|  | rs55764737 | -0.531 | 0.097 | -0.213 | 0.154 | 29.878 |
|  | rs57338032 | -0.158 | 0.032 | -0.012 | 0.050 | 24.937 |
|  | rs7333764 | 0.277 | 0.059 | -0.169 | 0.098 | 21.867 |
|  | rs76395525 | 0.528 | 0.108 | 0.273 | 0.181 | 23.742 |
| Eotaxin | rs112347425 | 0.158 | 0.028 | 0.030 | 0.065 | 32.535 |
|  | rs12075 | 0.167 | 0.016 | 0.002 | 0.037 | 114.737 |
|  | rs2024050 | -0.173 | 0.030 | 0.073 | 0.072 | 32.524 |
|  | rs2228467 | 0.416 | 0.029 | -0.065 | 0.070 | 203.258 |
| FGF-basic | rs13412535 | -0.111 | 0.023 | -0.033 | 0.521 | 24.426 |
|  | rs145577605 | 0.208 | 0.043 | 2.501 | 0.047 | 23.640 |
|  | rs747334 | -0.075 | 0.016 | 0.013 | 0.130 | 20.970 |
|  | rs75168112 | 0.100 | 0.021 | -0.095 | 0.037 | 21.880 |
|  | rs9907295 | -0.132 | 0.027 | 0.118 | 0.047 | 24.043 |
| G-CSF | rs115256310 | 0.682 | 0.136 | 0.018 | 0.215 | 25.155 |
|  | rs11903143 | -0.087 | 0.018 | -0.015 | 0.041 | 24.435 |
|  | rs147128865 | 0.270 | 0.059 | -0.002 | 0.127 | 21.157 |
|  | rs1817411 | 0.089 | 0.019 | -0.075 | 0.045 | 21.713 |
|  | rs2671444 | -0.078 | 0.017 | 0.040 | 0.039 | 22.306 |
|  | rs74148555 | -0.372 | 0.076 | 0.010 | 0.132 | 24.212 |
|  | rs76287671 | 0.094 | 0.019 | 0.034 | 0.044 | 24.631 |
|  | rs77318030 | 0.205 | 0.043 | -0.077 | 0.092 | 22.830 |
| GRO-a | rs12075 | 0.375 | 0.024 | 0.002 | 0.037 | 250.494 |
|  | rs185768063 | -0.400 | 0.076 | 0.048 | 0.116 | 27.673 |
|  | rs508977 | 0.380 | 0.028 | -0.014 | 0.044 | 184.378 |
| HGF | rs11060254 | -0.080 | 0.017 | -0.074 | 0.040 | 22.948 |
|  | rs150322232 | -0.210 | 0.046 | 0.006 | 0.097 | 20.650 |
|  | rs1698249 | 0.170 | 0.037 | 0.007 | 0.089 | 20.835 |
|  | rs2003620 | 0.228 | 0.049 | -0.099 | 0.111 | 21.721 |
|  | rs3748034 | 0.150 | 0.023 | -0.110 | 0.056 | 40.818 |
|  | rs5745687 | -0.307 | 0.041 | 0.194 | 0.093 | 57.252 |
|  | rs62481625 | -0.109 | 0.023 | 0.004 | 0.052 | 23.512 |
| IFN-γ | rs10487554 | -0.090 | 0.018 | -0.009 | 0.041 | 23.919 |
|  | rs113600793 | 0.183 | 0.037 | 0.042 | 0.080 | 24.044 |
|  | rs115729819 | -0.248 | 0.052 | -0.128 | 0.116 | 23.264 |
|  | rs11843756 | -0.184 | 0.039 | -0.061 | 0.092 | 21.921 |
|  | rs12420286 | -0.238 | 0.050 | 0.079 | 0.114 | 22.491 |
|  | rs1867282 | 0.077 | 0.017 | -0.046 | 0.038 | 21.740 |
|  | rs2073438 | 0.090 | 0.019 | 0.066 | 0.043 | 22.816 |
|  | rs74148555 | -0.373 | 0.077 | 0.010 | 0.132 | 23.249 |
|  | rs78296352 | 0.343 | 0.065 | 0.086 | 0.161 | 27.675 |
| IL-1rα | rs1054402 | -0.117 | 0.027 | 0.051 | 0.043 | 23.576 |
|  | rs11627423 | 0.269 | 0.025 | 0.029 | 0.039 | 22.476 |
|  | rs12121840 | 0.112 | 0.057 | 0.057 | 0.085 | 22.227 |
|  | rs56134659 | 0.445 | 0.024 | 0.059 | 0.037 | 22.213 |
|  | rs61335305 | -0.197 | 0.091 | -0.115 | 0.139 | 24.051 |
|  | rs9623661 | -0.131 | 0.043 | 0.064 | 0.066 | 21.298 |
| IL-1β | rs143319329 | 0.280 | 0.072 | 0.166 | 0.226 | 15.347 |
|  | rs1942793 | 0.072 | 0.019 | -0.111 | 0.037 | 14.701 |
|  | rs61335305 | 0.297 | 0.072 | -0.115 | 0.139 | 16.783 |
|  | rs62015704 | -0.108 | 0.028 | 0.057 | 0.057 | 14.618 |
|  | rs9898641 | 0.203 | 0.045 | 0.015 | 0.039 | 20.033 |
| IL-2 | rs12051139 | 0.113 | 0.025 | 0.038 | 0.038 | 20.967 |
|  | rs13412535 | 0.176 | 0.033 | -0.033 | 0.047 | 28.231 |
|  | rs170117 | -0.162 | 0.035 | 0.092 | 0.053 | 21.467 |
|  | rs2807544 | -0.118 | 0.025 | 0.064 | 0.039 | 21.569 |
|  | rs4634519 | 0.126 | 0.027 | -0.005 | 0.042 | 21.975 |
|  | rs61335305 | 0.451 | 0.092 | -0.115 | 0.139 | 24.179 |
|  | rs62124990 | -0.696 | 0.150 | -0.029 | 0.135 | 21.680 |
|  | rs7615304 | 0.117 | 0.024 | -0.009 | 0.037 | 23.454 |
|  | rs80336398 | -0.400 | 0.086 | -0.120 | 0.120 | 21.745 |
| IL-2rα | rs112352944 | -0.512 | 0.109 | 0.116 | 0.127 | 22.244 |
|  | rs114977391 | -0.625 | 0.125 | -0.138 | 0.187 | 25.065 |
|  | rs12617664 | 0.304 | 0.061 | -0.010 | 0.057 | 25.161 |
|  | rs12722497 | 0.869 | 0.056 | 0.021 | 0.074 | 241.952 |
|  | rs41367851 | 0.508 | 0.096 | -0.056 | 0.055 | 28.195 |
|  | rs62066672 | -0.299 | 0.060 | 0.072 | 0.053 | 24.885 |
|  | rs6510763 | 0.233 | 0.046 | 0.131 | 0.041 | 25.694 |
|  | rs71427735 | 0.221 | 0.046 | -0.039 | 0.048 | 22.765 |
|  | rs75881090 | -0.450 | 0.095 | 0.025 | 0.094 | 22.300 |
| IL-4 | rs10512267 | 0.082 | 0.016 | -0.031 | 0.038 | 26.194 |
|  | rs116705532 | 0.468 | 0.098 | 0.071 | 0.228 | 22.879 |
|  | rs117146485 | 0.292 | 0.063 | 0.024 | 0.140 | 21.610 |
|  | rs17713451 | 0.127 | 0.025 | -0.051 | 0.059 | 25.357 |
|  | rs73023729 | -0.180 | 0.037 | 0.168 | 0.091 | 24.080 |
|  | rs7613691 | -0.178 | 0.038 | 0.097 | 0.086 | 21.367 |
|  | rs79597994 | -0.583 | 0.127 | -0.190 | 0.226 | 21.080 |
|  | rs9508291 | 0.168 | 0.036 | 0.114 | 0.086 | 21.795 |
|  | rs9941733 | -0.114 | 0.023 | 0.012 | 0.049 | 24.782 |
| IL-5 | rs11680908 | -0.263 | 0.055 | 0.009 | 0.084 | 22.605 |
|  | rs6737109 | -0.116 | 0.025 | -0.050 | 0.037 | 22.056 |
|  | rs72831687 | -0.524 | 0.111 | 0.047 | 0.149 | 22.317 |
|  | rs73040130 | -0.264 | 0.053 | -0.010 | 0.082 | 24.868 |
|  | rs7767396 | -0.152 | 0.025 | 0.029 | 0.037 | 37.928 |
| IL-6 | rs1333040 | 0.074 | 0.016 | -0.028 | 0.037 | 21.817 |
|  | rs13412535 | -0.116 | 0.022 | -0.033 | 0.047 | 29.311 |
|  | rs72831623 | 0.197 | 0.037 | 0.055 | 0.074 | 28.130 |
|  | rs73273528 | 0.267 | 0.055 | -0.091 | 0.122 | 23.347 |
|  | rs76856708 | -0.329 | 0.070 | 0.190 | 0.161 | 22.077 |
| IL-7 | rs1891492 | -0.349 | 0.072 | 0.026 | 0.051 | 23.682 |
|  | rs305428 | -0.331 | 0.070 | -0.131 | 0.072 | 22.600 |
|  | rs6507581 | 0.309 | 0.064 | 0.073 | 0.052 | 22.984 |
|  | rs75219447 | -0.856 | 0.171 | 0.055 | 0.148 | 25.097 |
|  | rs767304 | 1.012 | 0.216 | 0.097 | 0.070 | 22.046 |
|  | rs8182389 | -0.920 | 0.192 | 0.158 | 0.169 | 22.883 |
| IL-8 | rs4887995 | -0.084 | 0.016 | 0.020 | 0.045 | 28.762 |
|  | rs6585420 | -0.163 | 0.032 | -0.062 | 0.091 | 26.375 |
|  | rs72886502 | -0.426 | 0.085 | -0.003 | 0.378 | 25.344 |
|  | rs7655660 | -0.188 | 0.024 | -0.154 | 0.094 | 62.657 |
|  | rs972492 | 0.111 | 0.020 | 0.117 | 0.063 | 30.087 |
| IL-9 | rs41294750 | 0.351 | 0.075 | 0.055 | 0.110 | 22.070 |
|  | rs4880409 | -0.336 | 0.072 | 0.081 | 0.186 | 21.533 |
|  | rs61867538 | 0.357 | 0.077 | 0.002 | 0.092 | 21.227 |
|  | rs7232268 | -0.276 | 0.059 | 0.075 | 0.095 | 22.092 |
|  | rs7242404 | -0.123 | 0.026 | -0.006 | 0.041 | 21.637 |
|  | rs76963786 | -0.287 | 0.056 | -0.111 | 0.089 | 26.457 |
| IL-10 | rs282258 | -0.099 | 0.016 | -0.003 | 0.038 | 37.497 |
|  | rs4349809 | -0.285 | 0.017 | 0.029 | 0.037 | 298.976 |
|  | rs7088799 | 0.085 | 0.017 | -0.001 | 0.038 | 26.028 |
| IL-12p70 | rs34467391 | 0.103 | 0.016 | -0.036 | 0.037 | 42.168 |
|  | rs4349809 | -0.378 | 0.016 | 0.029 | 0.037 | 564.287 |
|  | rs7088799 | 0.100 | 0.016 | -0.001 | 0.038 | 38.425 |
| IL-13 | rs117795020 | -0.352 | 0.072 | 0.117 | 0.111 | 24.197 |
|  | rs12623722 | -0.119 | 0.026 | -0.026 | 0.040 | 21.096 |
|  | rs139083458 | 0.990 | 0.211 | -0.071 | 0.305 | 22.086 |
|  | rs142167313 | 0.313 | 0.062 | 0.006 | 0.095 | 25.735 |
|  | rs27949 | -0.117 | 0.025 | -0.002 | 0.040 | 21.482 |
|  | rs6799107 | 0.146 | 0.030 | 0.000 | 0.048 | 23.495 |
|  | rs7073807 | -0.168 | 0.036 | -0.086 | 0.055 | 22.323 |
|  | rs75995699 | 0.332 | 0.070 | 0.282 | 0.104 | 22.610 |
|  | rs9472168 | -0.424 | 0.025 | 0.027 | 0.038 | 292.851 |
| IL-16 | rs1801020 | -0.173 | 0.027 | 0.026 | 0.026 | 40.594 |
|  | rs4253283 | -0.146 | 0.026 | 0.023 | 0.023 | 31.053 |
|  | rs4778636 | -0.727 | 0.063 | 0.109 | 0.109 | 131.978 |
| IL-17 | rs117029961 | 0.459 | 0.102 | -0.072 | 0.217 | 20.405 |
|  | rs117556572 | -0.510 | 0.110 | -0.120 | 0.124 | 21.552 |
|  | rs1530455 | -0.108 | 0.017 | -0.006 | 0.039 | 38.972 |
|  | rs17106604 | 0.113 | 0.023 | 0.133 | 0.052 | 25.178 |
|  | rs17282552 | 0.200 | 0.041 | -0.143 | 0.086 | 24.411 |
|  | rs184080173 | -0.238 | 0.047 | 0.122 | 0.106 | 25.620 |
|  | rs187475560 | -0.243 | 0.052 | 0.230 | 0.111 | 21.910 |
|  | rs62191444 | -0.114 | 0.025 | -0.004 | 0.052 | 21.153 |
|  | rs78296352 | 0.303 | 0.065 | 0.086 | 0.161 | 21.956 |
|  | rs78612928 | -0.104 | 0.022 | 0.062 | 0.050 | 21.820 |
| IL-18 | rs115267715 | 0.451 | 0.080 | -0.242 | 0.135 | 31.753 |
|  | rs17229943 | 0.312 | 0.046 | 0.142 | 0.062 | 45.410 |
|  | rs385076 | 0.243 | 0.025 | 0.033 | 0.039 | 96.166 |
|  | rs71478720 | -0.267 | 0.028 | -0.002 | 0.043 | 93.515 |
| IP-10 | rs10809307 | -0.131 | 0.028 | 0.023 | 0.045 | 21.415 |
|  | rs113831257 | 0.359 | 0.064 | 0.022 | 0.086 | 31.110 |
|  | rs11626201 | 0.116 | 0.025 | 0.036 | 0.039 | 22.495 |
|  | rs143799975 | 0.798 | 0.164 | -0.069 | 0.218 | 23.787 |
|  | rs34383175 | -0.315 | 0.066 | -0.066 | 0.103 | 23.031 |
|  | rs75970138 | -0.485 | 0.104 | 0.226 | 0.166 | 21.748 |
|  | rs7645625 | 0.109 | 0.024 | -0.056 | 0.038 | 20.997 |
|  | rs79848609 | -0.260 | 0.054 | -0.059 | 0.086 | 23.496 |
|  | rs8112909 | -0.143 | 0.030 | -0.044 | 0.047 | 22.746 |
|  | rs9450351 | 0.277 | 0.049 | -0.169 | 0.081 | 32.042 |
| MCP-1 | rs12075 | 0.219 | 0.016 | 0.002 | 0.037 | 198.719 |
|  | rs2036297 | -0.119 | 0.016 | 0.009 | 0.038 | 55.316 |
|  | rs2288370 | 0.103 | 0.016 | -0.018 | 0.039 | 40.008 |
|  | rs7632755 | 0.294 | 0.032 | 0.108 | 0.071 | 86.443 |
| MCP-3 | rs10892381 | 0.241 | 0.048 | 0.043 | 0.040 | 25.677 |
|  | rs62492260 | -0.279 | 0.058 | -0.057 | 0.054 | 23.106 |
|  | rs73669117 | 0.624 | 0.131 | 0.194 | 0.125 | 22.675 |
| M-CSF | rs116274860 | -0.819 | 0.174 | -0.082 | 0.165 | 22.129 |
|  | rs117867915 | -0.527 | 0.110 | 0.073 | 0.137 | 23.054 |
|  | rs12962919 | 0.305 | 0.066 | -0.083 | 0.073 | 21.255 |
|  | rs145778765 | -0.799 | 0.169 | -0.092 | 0.134 | 22.395 |
|  | rs56367447 | -0.497 | 0.088 | -0.147 | 0.105 | 31.642 |
|  | rs62294910 | 0.343 | 0.069 | 0.050 | 0.077 | 24.654 |
|  | rs78296352 | 0.527 | 0.111 | 0.086 | 0.161 | 22.460 |
|  | rs9387100 | 0.135 | 0.029 | 0.072 | 0.038 | 21.438 |
| MIF | rs12594190 | -0.136 | 0.027 | 0.032 | 0.041 | 25.755 |
|  | rs13142904 | -0.223 | 0.043 | 0.050 | 0.066 | 27.532 |
|  | rs78098071 | 0.487 | 0.092 | 0.153 | 0.147 | 28.108 |
| MIG | rs112861654 | 0.277 | 0.053 | 0.043 | 0.079 | 27.320 |
|  | rs1796086 | 0.210 | 0.040 | 0.064 | 0.067 | 27.050 |
|  | rs41272086 | -0.223 | 0.042 | 0.082 | 0.062 | 28.771 |
|  | rs55876513 | -0.166 | 0.026 | 0.051 | 0.041 | 42.378 |
| MIP-1α | rs10835056 | -0.119 | 0.025 | -0.006 | 0.040 | 22.097 |
|  | rs12690897 | 0.125 | 0.026 | -0.048 | 0.041 | 22.690 |
|  | rs184154340 | 0.331 | 0.069 | 0.045 | 0.096 | 22.813 |
|  | rs34771762 | -0.249 | 0.052 | -0.043 | 0.072 | 22.667 |
|  | rs57786342 | 0.131 | 0.029 | 0.107 | 0.049 | 21.257 |
|  | rs60198979 | -0.215 | 0.046 | -0.016 | 0.071 | 21.955 |
|  | rs7232268 | -0.282 | 0.060 | 0.075 | 0.095 | 22.180 |
| MIP-1β | rs113010081 | 0.595 | 0.024 | 0.015 | 0.055 | 636.493 |
|  | rs113877493 | -0.612 | 0.022 | -0.048 | 0.051 | 789.146 |
|  | rs117453826 | 0.577 | 0.059 | 0.061 | 0.125 | 94.808 |
|  | rs141102180 | 0.323 | 0.039 | -0.234 | 0.096 | 67.340 |
|  | rs17641689 | 0.245 | 0.029 | -0.045 | 0.060 | 69.805 |
|  | rs2079664 | -0.100 | 0.018 | 0.039 | 0.042 | 31.961 |
| PDGF-bb | rs13412535 | 0.335 | 0.021 | -0.033 | 0.047 | 245.347 |
|  | rs2324229 | -0.089 | 0.016 | 0.006 | 0.039 | 30.834 |
|  | rs4965869 | 0.184 | 0.018 | 0.137 | 0.043 | 103.342 |
|  | rs55680718 | -0.138 | 0.025 | -0.023 | 0.057 | 31.606 |
| RANTES | rs112072646 | 0.429 | 0.086 | -0.004 | 0.140 | 24.722 |
|  | rs147509526 | -0.358 | 0.072 | -0.094 | 0.107 | 24.930 |
|  | rs4940620 | 0.249 | 0.054 | 0.065 | 0.085 | 21.331 |
|  | rs62438851 | 0.196 | 0.041 | 0.005 | 0.061 | 22.345 |
|  | rs7000423 | -0.132 | 0.025 | -0.048 | 0.039 | 27.139 |
|  | rs72793342 | -0.149 | 0.031 | 0.086 | 0.046 | 23.309 |
|  | rs74472919 | 0.331 | 0.061 | -0.046 | 0.097 | 29.987 |
|  | rs75613039 | 0.370 | 0.081 | -0.112 | 0.115 | 20.866 |
|  | rs818452 | 0.238 | 0.051 | -0.072 | 0.077 | 22.230 |
| SCF | rs1557570 | 0.119 | 0.017 | -0.016 | 0.040 | 48.671 |
|  | rs1568119 | -0.591 | 0.113 | -0.369 | 0.254 | 27.365 |
|  | rs4841899 | 0.100 | 0.018 | 0.003 | 0.042 | 31.815 |
|  | rs635634 | -0.103 | 0.019 | 0.055 | 0.047 | 29.194 |
| SCGF-β | rs116924815 | 0.608 | 0.074 | -0.129 | 0.114 | 67.850 |
|  | rs117716477 | 0.838 | 0.084 | -0.046 | 0.149 | 99.383 |
|  | rs17876031 | 0.151 | 0.026 | 0.002 | 0.040 | 35.251 |
|  | rs181218758 | 0.383 | 0.059 | -0.119 | 0.102 | 42.784 |
|  | rs4656185 | 0.205 | 0.026 | -0.012 | 0.040 | 64.125 |
| SDF-1α | rs10474392 | -0.096 | 0.018 | -0.047 | 0.043 | 29.209 |
|  | rs12407262 | 0.118 | 0.027 | -0.063 | 0.060 | 19.646 |
|  | rs13400104 | 0.065 | 0.019 | -0.041 | 0.042 | 11.719 |
|  | rs139840550 | 0.183 | 0.055 | -0.087 | 0.135 | 11.160 |
|  | rs149893336 | 0.503 | 0.108 | 0.203 | 0.173 | 21.686 |
|  | rs4581824 | 0.070 | 0.017 | 0.057 | 0.039 | 16.419 |
|  | rs482700 | -0.089 | 0.020 | -0.046 | 0.047 | 19.351 |
|  | rs67689854 | -0.068 | 0.020 | 0.018 | 0.046 | 12.196 |
|  | rs9267091 | 0.078 | 0.020 | 1.870 | 0.056 | 14.802 |
| TNF-α | rs10834997 | -0.125 | 0.026 | 0.012 | 0.040 | 23.361 |
|  | rs111332265 | 0.377 | 0.075 | -0.039 | 0.102 | 24.947 |
|  | rs115669577 | 0.989 | 0.200 | -0.124 | 0.285 | 24.571 |
|  | rs79105320 | 0.561 | 0.118 | -0.244 | 0.174 | 22.601 |
|  | rs8121916 | 0.131 | 0.028 | -0.040 | 0.043 | 22.070 |
| TNF-β | rs10925040 | 0.176 | 0.037 | 0.009 | 0.039 | 22.138 |
|  | rs753274 | -0.174 | 0.037 | -0.011 | 0.038 | 21.895 |
|  | rs7629875 | -0.377 | 0.077 | -0.147 | 0.078 | 23.674 |
|  | rs78296352 | 1.222 | 0.137 | 0.086 | 0.161 | 79.962 |
| TRAIL | rs138987090 | 0.750 | 0.075 | 0.231 | 0.159 | 99.389 |
|  | rs193112415 | 1.042 | 0.062 | -0.133 | 0.135 | 279.797 |
|  | rs57396456 | 0.563 | 0.052 | -0.138 | 0.124 | 117.961 |
|  | rs62093514 | 1.062 | 0.055 | 0.016 | 0.128 | 370.005 |
|  | rs74778900 | 0.591 | 0.053 | 0.192 | 0.163 | 123.243 |
|  | rs79287178 | -0.432 | 0.042 | -0.010 | 0.095 | 105.148 |
| VEGF | rs13209117 | 0.130 | 0.020 | -0.004 | 0.043 | 41.959 |
|  | rs34467391 | 0.129 | 0.017 | -0.036 | 0.037 | 57.175 |
|  | rs6921438 | -0.490 | 0.018 | 0.025 | 0.037 | 784.000 |

Footnotes: β-NGF, beta nerve growth factor; CI, confidence interval; CTACK, cutaneous T-cell attracting (CCL27); FGF-basic, basic fibroblast growth factor; G-CSF, granulocyte colony-stimulating factor; GRO-a, growth regulated oncogene-α (CXCL1); HGF, hepatocyte growth factor; IFN-γ, interferon-gamma; IL-1rα, interleukin-1 receptor antagonist; IL-1β, interleukin-1-beta; IL-2, interleukin-2; IL-2rα, interleukin-2 receptor, alpha subunit; IL-4, interleukin-4; IL-5, interleukin-5; IL-6, interleukin-6; IL-7, interleukin-7; IL-8, interleukin-8; IL-9, interleukin-9; IL-10, interleukin-10; IL-12p70, interleukin-12p70; IL-13, interleukin-13; IL-16, interleukin-16; IL-17, interleukin-17; IL-18, interleukin-18; IP-10, interferon gamma-induced protein 10 (CXCL10); MCP-1, monocyte chemotactic protein-1; MCP-3, monocyte specific chemokine 3 (CCL7); M-CSF, macrophage colony-stimulating factor; MIF, macrophage migration inhibitory factor; MIG, monokine induced by interferon-gamma; MIP-1α, macrophage inflammatory protein-1α (CCL3); MIP-1b, macrophage inflammatory protein-1β; PDGF-bb, platelet derived growth factor BB; RANTES, regulated on activation normal T Cell expressed and secreted (CCL5); SCF, stem cell factor; SCGF-β, stem cell growth factor beta; SDF-1α, stromal cell-derived factor-1 alpha; SNP, single nucleotide polymorphism; TNF-α, tumor necrosis factor-alpha; TNF-β, tumor necrosis factor-beta; TRAIL, TNF-related apoptosis inducing ligand; VEGF, vascular endothelial growth factor.

**Table S3.** Characteristics of the genetic variants associated with the ankylosing spondylitis.

| SNP | Chr | Effect allele | Beta | SE | *F*-statistic | *P*-value |
| --- | --- | --- | --- | --- | --- | --- |
| rs10807943 | 7 | C | -0.563 | 0.081 | 48.039 | 4.10×10^-12^ |
| rs112733823 | 6 | T | 0.360 | 0.047 | 59.172 | 1.43×10^-14^ |
| rs13033284 | 2 | C | -0.221 | 0.039 | 32.899 | 9.67×10^-09^ |
| rs16894011 | 6 | A | 2.108 | 0.089 | 565.806 | 5.32×10^-125^ |
| rs181316459 | 7 | C | 0.985 | 0.100 | 96.192 | 1.02×10^-22^ |
| rs34982906 | 6 | C | 0.821 | 0.092 | 80.003 | 3.82×10^-19^ |
| rs62394289 | 6 | A | 0.374 | 0.056 | 45.037 | 1.90×10^-11^ |
| rs76644067 | 6 | A | 0.733 | 0.095 | 60.005 | 9.44×10^-15^ |
| rs79693223 | 6 | T | 1.295 | 0.106 | 148.762 | 3.36×10^-34^ |
| rs9264277 | 6 | C | 0.520 | 0.045 | 134.622 | 3.73×10^-31^ |
| rs9265893 | 6 | C | 1.943 | 0.059 | 1091.471 | 1.00×10^-200^ |
| rs9378220 | 6 | A | -0.693 | 0.056 | 154.374 | 1.97×10^-35^ |
| rs9391773 | 6 | T | 2.635 | 0.070 | 1408.815 | 1.00×10^-200^ |

Footnotes: Chr, chromosome; SE, standard error; SNP, single nucleotide polymorphism.

**Table S4.** Details of the number of genetic instruments, Variance explained and *F*-statistic for each cytokine.

| Abbreviations | No. of SNPs | *R*²† | *F*-statistic (range) ‡ |  |
| --- | --- | --- | --- | --- |
| β-NGF | 7 | 0.007 | 24.166（20.765 - 36.504） |  |
| CTACK | 9 | 0.010 | 37.259 (22.432 - 142.656) |  |
| Eotaxin | 4 | 0.010 | 95.764 (32.524 - 203.258) |  |
| FGF-basic | 5 | 0.003 | 22.992 (20.970 - 24.426) |  |
| G-CSF | 8 | 0.003 | 23.305 (21.157 - 25.155) |  |
| GRO-a | 3 | 0.041 | 154.182 (27.673 - 250.494) |  |
| HGF | 7 | 0.004 | 29.677 (20.650 - 57.252) |  |
| IFN-γ | 9 | 0.003 | 23.458 (21.740 - 27.675) |  |
| IL-1rα | 6 | 0.006 | 22.640 (21.298 - 24.051) |  |
| IL-1β | 5 | 0.005 | 16.296(14.618 - 20.033) |  |
| IL-2 | 9 | 0.007 | 22.807 (20.967 - 28.231) |  |
| IL-2rα | 9 | 0.033 | 48.696 (22.244 - 241.952) |  |
| IL-4 | 9 | 0.003 | 23.238 (21.08 - 26.194) |  |
| IL-5 | 5 | 0.008 | 25.955 (22.056 - 37.928) |  |
| IL-6 | 5 | 0.003 | 24.936 (21.817 - 29.311) |  |
| IL-7 | 6 | 0.023 | 23.215 (22.046 - 25.097) |  |
| IL-8 | 5 | 0.002 | 34.645 (25.344 - 62.657) |  |
| IL-9 | 6 | 0.006 | 22.503 (21.227 - 26.457) |  |
| IL-10 | 3 | 0.015 | 120.834 (26.028 - 298.976) |  |
| IL-12p70 | 3 | 0.024 | 214.960 (38.425 - 564.287) |  |
| IL-13 | 9 | 0.014 | 52.875 (21.096 - 292.851) |  |
| IL-16 | 3 | 0.019 | 67.875 (31.053 - 131.978) |  |
| IL-17 | 10 | 0.003 | 24.298 (20.405 - 38.972) |  |
| IL-18 | 4 | 0.018 | 66.711 (31.753 - 96.166) |  |
| IP-10 | 10 | 0.007 | 24.287 (20.997 - 32.042) |  |
| MCP-1 | 4 | 0.011 | 95.122 (40.008 - 198.719) |  |
| MCP-3 | 3 | 0.027 | 23.819 (22.675 - 25.677) |  |
| M-CSF | 8 | 0.027 | 23.628 (21.255 - 31.642) |  |
| MIF | 3 | 0.008 | 27.132 (25.755 - 28.108) |  |
| MIG | 4 | 0.008 | 31.380 (27.050 - 42.378) |  |
| MIP-1α | 7 | 0.006 | 22.237 (21.257 - 22.813) |  |
| MIP-1b | 6 | 0.031 | 281.592 (31.961 - 789.146) |  |
| PDGF-bb | 4 | 0.012 | 102.782 (30.834 - 245.347) |  |
| RANTES | 9 | 0.007 | 24.095 (20.866 - 29.987) |  |
| SCF | 4 | 0.004 | 34.261 (27.365 - 48.671) |  |
| SCGF-β | 5 | 0.016 | 61.879 (35.251 - 99.383) |  |
| SDF-1α | 9 | 0.003 | 17.354 (11.160 - 29.209) |  |
| TNF-α | 5 | 0.007 | 23.510 (22.070 - 24.947) |  |
| TNF-β | 4 | 0.023 | 36.917 (21.895 - 79.962) |  |
| TRAIL | 6 | 0.022 | 182.591 (99.389 - 370.005) |  |
| VEGF | 3 | 0.036 | 294.378 (41.959 - 784.000) |  |

Footnotes: β-NGF, beta nerve growth factor; CI, confidence interval; CTACK, cutaneous T-cell attracting (CCL27); FGF-basic, basic fibroblast growth factor; G-CSF, granulocyte colony-stimulating factor; GRO-a, growth regulated oncogene-α (CXCL1); HGF, hepatocyte growth factor; IFN-γ, interferon-gamma; IL-1rα, interleukin-1 receptor antagonist; IL-1β, interleukin-1-beta; IL-2, interleukin-2; IL-2rα, interleukin-2 receptor, alpha subunit; IL-4, interleukin-4; IL-5, interleukin-5; IL-6, interleukin-6; IL-7, interleukin-7; IL-8, interleukin-8; IL-9, interleukin-9; IL-10, interleukin-10; IL-12p70, interleukin-12p70; IL-13, interleukin-13; IL-16, interleukin-16; IL-17, interleukin-17; IL-18, interleukin-18; IP-10, interferon gamma-induced protein 10 (CXCL10); MCP-1, monocyte chemotactic protein-1; MCP-3, monocyte specific chemokine 3 (CCL7); M-CSF, macrophage colony-stimulating factor; MIF, macrophage migration inhibitory factor; MIG, monokine induced by interferon-gamma; MIP-1α, macrophage inflammatory protein-1α (CCL3); MIP-1b, macrophage inflammatory protein-1β; MR, Mendelian randomization; No., number; OR, odds ratio; PDGF-bb, platelet derived growth factor BB; RANTES, regulated on activation normal T Cell expressed and secreted (CCL5); SCF, stem cell factor; SCGF-β, stem cell growth factor beta; SDF-1α, stromal cell-derived factor-1 alpha; SNP, single nucleotide polymorphism; TNF-α, tumor necrosis factor-alpha; TNF-β, tumor necrosis factor-beta; TRAIL, TNF-related apoptosis inducing ligand; VEGF, vascular endothelial growth factor.

†*R*² (Variance explained) = Beta² / (Beta²+SE²×N); Beta, the effect estimate of the SNP; SE, standard error; N, sample size.

‡ *F =* Beta² / SE², where Beta is the effect estimate of the SNP and SE its standard error on the respective cytokine levels.

**Table S5.** Characteristics of instrumental variables used for circulating levels of CTACK and MCP-3 in this study.

| Cytokines/ Growth factors | SNP | Chr | Position | Effect allele | Beta | SE | *P*-value |
| --- | --- | --- | --- | --- | --- | --- | --- |
| CTACK | rs116303454 | 3 | 27294655 | A | 0.383 | 0.082 | 3.27×10^-6^ |
| CTACK | rs145902143 | 12 | 20954590 | G | 0.284 | 0.058 | 1.03×10^-6^ |
| CTACK | rs2070074 | 9 | 34649442 | G | -0.447 | 0.037 | 1.78×10^-32^ |
| CTACK | rs2731674 | 5 | 176839890 | G | 0.133 | 0.027 | 5.63×10^-7^ |
| CTACK | rs3766110 | 1 | 169515183 | C | 0.129 | 0.028 | 3.85×10^-6^ |
| CTACK | rs55764737 | 15 | 61323414 | C | -0.531 | 0.097 | 4.62×10^-8^ |
| CTACK | rs57338032 | 15 | 78798939 | G | -0.158 | 0.032 | 6.23×10^-7^ |
| CTACK | rs7333764 | 13 | 34208801 | T | 0.277 | 0.059 | 2.85×10^-6^ |
| CTACK | rs76395525 | 15 | 79741391 | A | 0.528 | 0.108 | 9.55×10^-7^ |
| MCP-3 | rs10892381 | 11 | 119400817 | T | 0.241 | 0.048 | 3.56×10^-7^ |
| MCP-3 | rs62492260 | 8 | 10476528 | T | -0.279 | 0.058 | 1.54×10^-6^ |
| MCP-3 | rs73669117 | 8 | 27835190 | G | 0.624 | 0.131 | 2.56×10^-6^ |

Footnotes: Chr, chromosome; CTACK, Cutaneous T-cell attracting (CCL27); MCP-3, Monocyte specific chemokine 3 (CCL7); SE, standard error; SNP, single nucleotide polymorphism.

**Table S6.** Effect estimates of the associations between circulating levels of 41 cytokines and risk of ankylosing spondylitis in MR analyses.

| Methods | No. of SNPs | OR | 95% CI | *P-*value |
| --- | --- | --- | --- | --- |
| β-NGF |  |  |  |  |
| Inverse-variance weighted | 7** | 0.830 | 0.651 - 1.057 | 0.130 |
| Weighted median | 7** | 0.921 | 0.673 - 1.260 | 0.606 |
| MR-Egger | 7** | 0.580 | 0.165 - 2.042 | 0.435 |
| CTACK |  |  |  |  |
| Inverse-variance weighted | 9** | 1.224 | 1.033 - 1.451 | 0.020 |
| Weighted median | 9** | 1.256 | 1.024 - 1.589 | 0.030 |
| MR-Egger | 9** | 1.371 | 0.981 - 1.914 | 0.107 |
| Eotaxin |  |  |  |  |
| Inverse-variance weighted | 4 | 0.906 | 0.714 - 1.151 | 0.419 |
| Weighted median | 4 | 0.910 | 0.696 - 1.189 | 0.489 |
| MR-Egger | 4 | 0.777 | 0.430 - 1.406 | 0.493 |
| FGF-basic |  |  |  |  |
| Inverse-variance weighted | 5** | 2.964 | 0.055 - 1.588e+02 | 0.593 |
| Weighted median | 5** | 0.691 | 0.382 - 1.249 | 0.220 |
| MR-Egger | 5** | 3375 | 0.037 - 3.105e+10 | 0.223 |
| G-CSF |  |  |  |  |
| Inverse-variance weighted | 8** | 0.901 | 0.671 - 1.208 | 0.485 |
| Weighted median | 8** | 0.993 | 0.679 - 1.452 | 0.969 |
| MR-Egger | 8** | 1.016 | 0.612 - 1.685 | 0.953 |
| GRO-α |  |  |  |  |
| Inverse-variance weighted | 3* | 0.980 | 0.850 - 1.130 | 0.782 |
| Weighted median | 3* | 0.984 | 0.847 - 1.143 | 0.833 |
| MR-Egger | 3* | 0.118 | 0.001 - 1375 | 0.732 |
| HGF |  |  |  |  |
| Inverse-variance weighted | 7** | 0.767 | 0.515 - 1.143 | 0.192 |
| Weighted median | 7** | 0.644 | 0.418 - 0.991 | 0.045 |
| MR-Egger | 7** | 0.341 | 0.164 - 0.712 | 0.035 |
| IFN-γ |  |  |  |  |
| Inverse-variance weighted | 9** | 1.136 | 0.813 - 1.750 | 0.397 |
| Weighted median | 9** | 1.193 | 0.890 - 1.240 | 0.367 |
| MR-Egger | 9** | 1.211 | 0.677 - 2.167 | 0.540 |
| IL-1rα |  |  |  |  |
| Inverse-variance weighted | 6** | 0.922 | 0.690 - 1.231 | 0.580 |
| Weighted median | 6** | 0.775 | 0.552 - 1.088 | 0.141 |
| MR-Egger | 6** | 0.787 | 0.352 - 1.757 | 0.591 |
| IL-1β |  |  |  |  |
| Inverse-variance weighted | 5** | 0.854 | 0.522 - 1.395 | 0.527 |
| Weighted median | 5** | 0.950 | 0.655 - 1.378 | 0.788 |
| MR-Egger | 5** | 2.092 | 1.046 - 4.188 | 0.128 |
| IL-2 |  |  |  |  |
| Inverse-variance weighted | 9** | 0.919 | 0.760 - 1.111 | 0.382 |
| Weighted median | 9** | 0.955 | 0.735 - 1.242 | 0.734 |
| MR-Egger | 9** | 1.035 | 0.706 - 1.518 | 0.864 |
| IL2ra |  |  |  |  |
| Inverse-variance weighted | 9** | 0.994 | 0.865 - 1.141 | 0.929 |
| Weighted median | 9** | 0.967 | 0.844 - 1.107 | 0.626 |
| MR-Egger | 9** | 0.915 | 0.665 - 1.261 | 0.605 |
| IL-4 |  |  |  |  |
| Inverse-variance weighted | 9** | 0.899 | 0.661 - 1.222 | 0.496 |
| Weighted median | 9** | 0.933 | 0.615 - 1.414 | 0.743 |
| MR-Egger | 9** | 1.276 | 0.689 - 2.366 | 0.464 |
| IL-5 |  |  |  |  |
| Inverse-variance weighted | 5** | 1.000 | 0.775 - 1.291 | 1.000 |
| Weighted median | 5** | 0.945 | 0.682 - 1.308 | 0.734 |
| MR-Egger | 5** | 0.809 | 0.441 - 1.481 | 0.541 |
| IL-6 |  |  |  |  |
| Inverse-variance weighted | 5** | 0.933 | 0.636 - 1.369 | 0.724 |
| Weighted median | 5** | 0.941 | 0.588 - 1.506 | 0.800 |
| MR-Egger | 5** | 0.872 | 0.366 - 2.074 | 0.776 |
| IL-7 |  |  |  |  |
| Inverse-variance weighted | 6** | 1.071 | 0.953 - 1.204 | 0.248 |
| Weighted median | 6** | 1.057 | 0.926 - 1.206 | 0.412 |
| MR-Egger | 6** | 1.001 | 0.779 - 1.287 | 0.992 |
| IL-8 |  |  |  |  |
| Inverse-variance weighted | 5* | 1.579 | 0.955 - 2.612 | 0.075 |
| Weighted median | 5* | 1.639 | 0.870 - 3.086 | 0.126 |
| MR-Egger | 5* | 2.482 | 0.604 - 10.187 | 0.296 |
| IL-9 |  |  |  |  |
| Inverse-variance weighted | 6** | 1.057 | 0.814 - 1.372 | 0.679 |
| Weighted median | 6** | 1.040 | 0.746 - 1.450 | 0.816 |
| MR-Egger | 6** | 1.060 | 0.577 - 1.950 | 0.860 |
| IL-10 |  |  |  |  |
| Inverse-variance weighted | 3* | 0.920 | 0.728 - 1.162 | 0.484 |
| Weighted median | 3* | 0.918 | 0.716 - 1.176 | 0.497 |
| MR-Egger | 3* | 0.855 | 0.537 - 1.363 | 0.630 |
| IL-12p70 |  |  |  |  |
| Inverse-variance weighted | 3 | 0.912 | 0.761 - 1.094 | 0.321 |
| Weighted median | 3 | 0.924 | 0.762 - 1.119 | 0.418 |
| MR-Egger | 3 | 0.962 | 0.695 - 1.332 | 0.856 |
| IL-13 |  |  |  |  |
| Inverse-variance weighted | 9** | 1.018 | 0.862 - 1.203 | 0.833 |
| Weighted median | 9** | 0.954 | 0.810 - 1.123 | 0.572 |
| MR-Egger | 9** | 0.897 | 0.649 - 1.239 | 0.531 |
| IL-16 |  |  |  |  |
| Inverse-variance weighted | 3 | 0.860 | 0.704 - 1.050 | 0.139 |
| Weighted median | 3 | 0.861 | 0.699 - 1.059 | 0.157 |
| MR-Egger | 3 | 0.862 | 0.624 - 1.192 | 0.534 |
| IL-17 |  |  |  |  |
| Inverse-variance weighted | 10** | 0.950 | 0.670 - 1.346 | 0.773 |
| Weighted median | 10** | 1.048 | 0.738 - 1.487 | 0.792 |
| MR-Egger | 10** | 0.897 | 0.435 - 1.848 | 0.775 |
| IL-18 |  |  |  |  |
| Inverse-variance weighted | 4 | 1.101 | 0.814 - 1.490 | 0.531 |
| Weighted median | 4 | 1.097 | 0.874 - 1.377 | 0.426 |
| MR-Egger | 4 | 0.578 | 0.064 - 5.248 | 0.675 |
| IP-10 |  |  |  |  |
| Inverse-variance weighted | 10** | 0.933 | 0.755 - 1.153 | 0.520 |
| Weighted median | 10** | 1.007 | 0.767 - 1.322 | 0.960 |
| MR-Egger | 10** | 0.886 | 0.571 - 1.374 | 0.603 |
| MCP-1 |  |  |  |  |
| Inverse-variance weighted | 4 | 1.068 | 0.842 - 1.354 | 0.588 |
| Weighted median | 4 | 1.008 | 0.751 - 1.352 | 0.959 |
| MR-Egger | 4 | 1.515 | 0.797 - 2.882 | 0.333 |
| MCP-3 |  |  |  |  |
| Inverse-variance weighted | 3** | 1.250 | 1.016 - 1.539 | 0.035 |
| Weighted median | 3** | 1.220 | 0.948 - 1.571 | 0.123 |
| MR-Egger | 3** | 1.481 | 0.756 - 2.901 | 0.457 |
| M-CSF |  |  |  |  |
| Inverse-variance weighted | 8** | 1.120 | 0.959 - 1.309 | 0.152 |
| Weighted median | 8** | 1.127 | 0.913 - 1.390 | 0.265 |
| MR-Egger | 8** | 0.993 | 0.756 - 1.306 | 0.964 |
| MIF |  |  |  |  |
| Inverse-variance weighted | 3* | 0.951 | 0.666 - 1.356 | 0.780 |
| Weighted median | 3* | 0.798 | 0.505 - 1.260 | 0.333 |
| MR-Egger | 3* | 1.512 | 0.679 - 3.367 | 0.496 |
| MIG |  |  |  |  |
| Inverse-variance weighted | 4* | 0.909 | 0.662 - 1.250 | 0.558 |
| Weighted median | 4* | 0.861 | 0.618 - 1.199 | 0.375 |
| MR-Egger | 4* | 1.978 | 0.332 - 11.772 | 0.532 |
| MIP-1α |  |  |  |  |
| Inverse-variance weighted | 7** | 1.073 | 0.828 - 1.390 | 0.594 |
| Weighted median | 7** | 1.089 | 0.789 - 1.501 | 0.604 |
| MR-Egger | 7** | 1.035 | 0.478 - 2.241 | 0.933 |
| MIP-1β |  |  |  |  |
| Inverse-variance weighted | 6 | 1.010 | 0.875 - 1.166 | 0.890 |
| Weighted median | 6 | 1.054 | 0.928 - 1.196 | 0.414 |
| MR-Egger | 6 | 1.186 | 0.931 - 1.509 | 0.239 |
| PDGF-bb |  |  |  |  |
| Inverse-variance weighted | 4 | 1.121 | 0.757 - 1.662 | 0.568 |
| Weighted median | 4 | 0.922 | 0.697 - 1.221 | 0.574 |
| MR-Egger | 4 | 0.892 | 0.333 - 2.391 | 0.841 |
| RANTES |  |  |  |  |
| Inverse-variance weighted | 9** | 0.956 | 0.777 - 1.176 | 0.671 |
| Weighted median | 9** | 0.993 | 0.746 - 1.321 | 0.960 |
| MR-Egger | 9** | 0.934 | 0.525 - 1.661 | 0.824 |
| SCF |  |  |  |  |
| Inverse-variance weighted | 4* | 0.992 | 0.642 - 1.532 | 0.970 |
| Weighted median | 4* | 0.938 | 0.575 - 1.532 | 0.799 |
| MR-Egger | 4* | 2.188 | 0.787 - 6.082 | 0.272 |
| SCGF-β |  |  |  |  |
| Inverse-variance weighted | 5 | 0.889 | 0.741 - 1.068 | 0.209 |
| Weighted median | 5 | 0.945 | 0.763 - 1.170 | 0.602 |
| MR-Egger | 5 | 0.827 | 0.592 - 1.156 | 0.347 |
| SDF-1α |  |  |  |  |
| Inverse-variance weighted | 9** | 4.914 | 0.098 - 246.29 | 0.425 |
| Weighted median | 9** | 1.511 | 0.925 - 2.466 | 0.099 |
| MR-Egger | 9** | 0.679 | 0.0001 - 4081 | 0.933 |
| TNF-α |  |  |  |  |
| Inverse-variance weighted | 5** | 0.816 | 0.627 - 1.064 | 0.133 |
| Weighted median | 5** | 0.885 | 0.635 - 1.235 | 0.474 |
| MR-Egger | 5** | 0.806 | 0.516 - 1.257 | 0.411 |
| TNF-β |  |  |  |  |
| Inverse-variance weighted | 4** | 1.135 | 0.950 - 1.355 | 0.163 |
| Weighted median | 4** | 1.071 | 0.865 - 1.326 | 0.527 |
| MR-Egger | 4** | 1.151 | 0.860 - 1.539 | 0.444 |
| TRAIL |  |  |  |  |
| Inverse-variance weighted | 6 | 1.000 | 0.864 - 1.158 | 0.997 |
| Weighted median | 6 | 0.989 | 0.831 - 1.176 | 0.899 |
| MR-Egger | 6 | 0.938 | 0.586 - 1.503 | 0.804 |
| VEGF |  |  |  |  |
| Inverse-variance weighted | 3 | 0.938 | 0.815 - 1.080 | 0.374 |
| Weighted median | 3 | 0.947 | 0.817 - 1.099 | 0.474 |
| MR-Egger | 3 | 0.993 | 0.770 - 1.280 | 0.965 |

Footnotes: β-NGF, beta nerve growth factor; CI, confidence interval; CTACK, cutaneous T-cell attracting (CCL27); FGF-basic, basic fibroblast growth factor; G-CSF, granulocyte colony-stimulating factor; GRO-a, growth regulated oncogene-α (CXCL1); HGF, hepatocyte growth factor; IFN-γ, interferon-gamma; IL-1rα, interleukin-1 receptor antagonist; IL-1β, interleukin-1-beta; IL-2, interleukin-2; IL-2rα, interleukin-2 receptor, alpha subunit; IL-4, interleukin-4; IL-5, interleukin-5; IL-6, interleukin-6; IL-7, interleukin-7; IL-8, interleukin-8; IL-9, interleukin-9; IL-10, interleukin-10; IL-12p70, interleukin-12p70; IL-13, interleukin-13; IL-16, interleukin-16; IL-17, interleukin-17; IL-18, interleukin-18; IP-10, interferon gamma-induced protein 10 (CXCL10); MCP-1, monocyte chemotactic protein-1; MCP-3, monocyte specific chemokine 3 (CCL7); M-CSF, macrophage colony-stimulating factor; MIF, macrophage migration inhibitory factor; MIG, monokine induced by interferon-gamma; MIP-1α, macrophage inflammatory protein-1α (CCL3); MIP-1b, macrophage inflammatory protein-1β; MR, Mendelian randomization; OR, odds ratio; PDGF-bb, platelet derived growth factor BB; RANTES, regulated on activation normal T Cell expressed and secreted (CCL5); SCF, stem cell factor; SCGF-β, stem cell growth factor beta; SDF-1α, stromal cell-derived factor-1 alpha; SNP, single nucleotide polymorphism; TNF-α, tumor necrosis factor-alpha; TNF-β, tumor necrosis factor-beta; TRAIL, TNF-related apoptosis inducing ligand; VEGF, vascular endothelial growth factor.

*: Number of SNPs with significance threshold at *P* < 5 × 10^-7^; **: Number of SNPs with significance threshold at *P* < 5 × 10^-6^.

**Table S7.** Details of the genetic variants with potential pleiotropy among instrumental variables of CTACK.

| Cytokines | SNP | Pleiotropic trait^*^ | *P*-value | PMID |
| --- | --- | --- | --- | --- |
| CTACK | rs2731674 | Serum levels of protein APLP2 | 8.00 x 10^-12^ | 35078996 |
|  |  | Serum levels of protein PTHLH | 4.00 x 10^-18^ | 35078996 |
|  |  | Serum levels of protein ARL1 | 6.00. x 10^-12^ | 35078996 |
|  |  | Serum levels of protein F9 | 2.00 x 10^-63^ | 35078996 |
|  |  | Serum levels of protein F9 | 3.00 x 10^-46^ | 35078996 |
|  |  | Serum levels of protein ANP32B | 4.00 x 10^-21^ | 35078996 |
|  |  | Serum levels of protein ALDH1A3 | 7.00 x 10^-18^ | 35078996 |
|  |  | Serum levels of protein RAET1L | 5.00 x 10^-17^ | 35078996 |
|  |  | Serum levels of protein THBS1 | 3.00 x 10^-14^ | 35078996 |
|  |  | Serum levels of protein TPMT | 2.00 x 10^-13^ | 35078996 |
|  |  | Serum levels of protein CD63 | 1.00 x 10^-19^ | 35078996 |
|  |  | Serum levels of protein LGALS7 | 7.00 x 10^-18^ | 35078996 |
|  |  | Serum levels of protein SERPINA5 | 1.00 x 10^-41^ | 35078996 |
|  |  | Serum levels of protein SOD2 | 2.00 x 10^-151^ | 35078996 |
|  |  | Serum levels of protein MRPL58 | 1.00 x 10^-45^ | 35078996 |
|  |  | Serum levels of protein LEAP2 | 2.00 x 10^-104^ | 35078996 |
|  |  | Serum levels of protein DEFB113 | 4.00 x 10^-64^ | 35078996 |
|  |  | Serum levels of protein CFC1 | 1.00 x 10^-25^ | 35078996 |
|  |  | Serum levels of protein KLKB1 | 6.00 x 10^-23^ | 35078996 |
|  |  | Progonadoliberin-1 levels | 9.00 x 10^-13^ | 29875488 |
|  |  | Tumor necrosis factor receptor superfamily member 16 levels | 3.00 x 10^-14^ | 29875488 |
|  |  | Activating signal cointegrator 1 complex subunit 1 levels | 2.00 x 10^-15^ | 29875488 |
|  |  | Parathyroid hormone-related protein levels | 2.00 x 10^-13^ | 29875488 |

Footnotes: PMID, PubMed Unique Identifier; SNP, single nucleotide polymorphism.

^*^ From the Phenoscanner Database (version 2) (http://www.phenoscanner.medschl.cam.ac.uk, last accessed on Aug 20th, 2023)

**Table S8.** Effect estimates for the association of circulating CTACK levels with risk of ankylosing spondylitis after exclusion of potential multidirectional SNPs.

| Methods | No. of SNPs | OR | 95% CI | *P-*value |
| --- | --- | --- | --- | --- |
| CTACK | | | | |
| Inverse-variance weighted | 8 | 1.224 | 1.022 - 1.468 | 0.028 |
| Weighted median | 8 | 1.277 | 1.014 - 1.606 | 0.037 |
| MR-Egger | 8 | 1.425 | 0.979 - 2.074 | 0.114 |

Footnotes: CI, confidence interval; CTACK, cutaneous T-cell attracting (CCL27); MR, Mendelian randomization; No., number; OR, odds ratio; SNP, single nucleotide polymorphism.

**Table S9.** Effect estimates of the associations of ankylosing spondylitis with risk of circulating levels of FGF-basic, G-CSF and MCP-3.

| Methods | No. of SNPs | Beta | 95% CI | *P-*value |
| --- | --- | --- | --- | --- |
| FGF-basic | | | | |
| Inverse-variance weighted | 10 | 0.023 | 0.004 - 0.042 | 0.017 |
| Weighted median | 10 | 0.025 | 0.007 - 0.043 | 0.005 |
| MR-Egger | 10 | 0.030 | 0.002 - 0.057 | 0.075 |
| G-CSF | | | | |
| Inverse-variance weighted | 10 | 0.017 | 0.002 - 0.032 | 0.025 |
| Weighted median | 10 | 0.015 | -0.001 - 0.031 | 0.082 |
| MR-Egger | 10 | 0.017 | -0.004 - 0.039 | 0.157 |
| MCP-3 | | | | |
| Inverse-variance weighted | 9 | 0.053 | 0.007 - 0.099 | 0.025 |
| Weighted median | 9 | 0.040 | -0.005 - 0.085 | 0.099 |
| MR-Egger | 9 | 0.039 | -0.031 - 0.106 | 0.316 |

Footnotes: Beta, effect estimate; CI, confidence interval; FGF-basic, basic fibroblast growth factor; G-CSF, Granulocyte colony-stimulating factor; MCP-3, Monocyte specific chemokine 3 (CCL7); MR, Mendelian randomization; No., number; SNP, single nucleotide polymorphism.

**Figure S1.** MR Plots for Relationship of CTACK with AS.

**
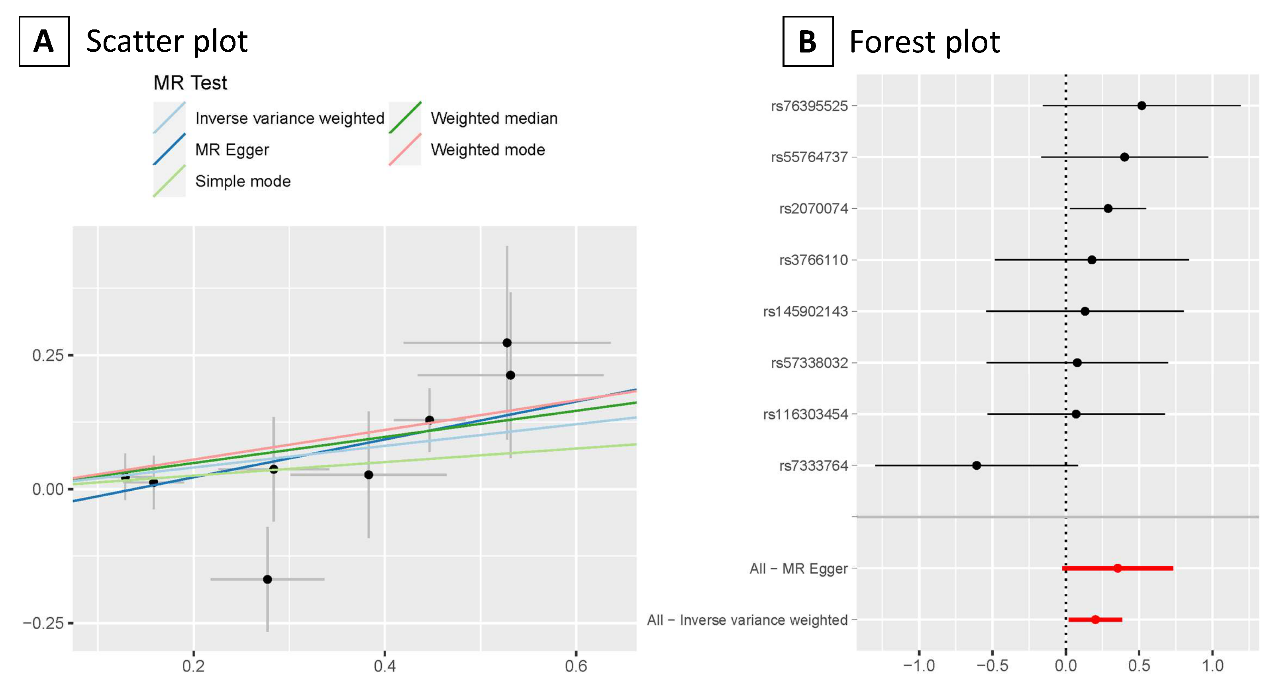
**

Footnotes: AS, ankylosing spondylitis; CTACK, cutaneous T-cell attracting (CCL27); MR, mendelian randomization; A, Scatter plot of single-nucleotide polymorphism (SNP) effects on CTACK vs AS, with the slope of each line corresponding to estimated MR effect per method. B, Forest plot of individual and combined SNP MR-estimated effect sizes.

**Figure S2.** MR Plots for Relationship of MCP-3 with AS.

**
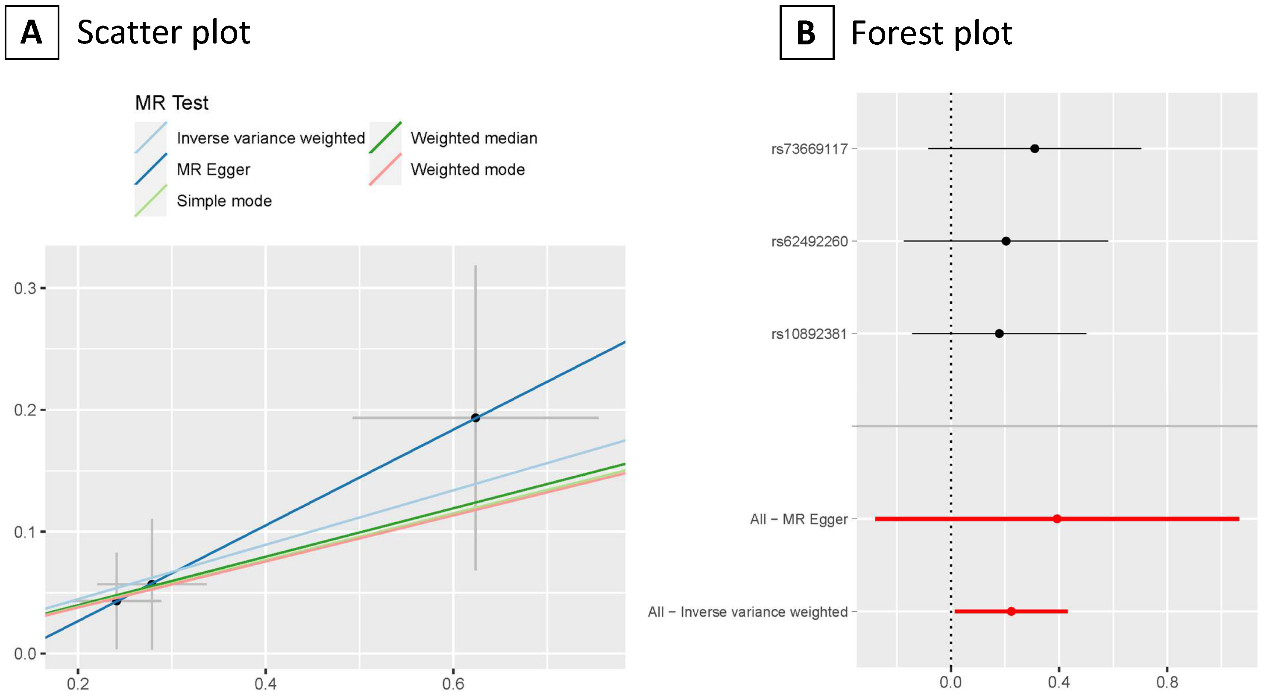
**

Footnotes: AS, ankylosing spondylitis; MCP-3, Monocyte specific chemokine 3 (CCL7); MR, mendelian randomization; A, Scatter plot of single-nucleotide polymorphism (SNP) effects on MCP-3 vs AS, with the slope of each line corresponding to estimated MR effect per method. B, Forest plot of individual and combined SNP MR-estimated effect sizes.

**Figure S3.** MR Plots for Relationship of AS with FGF-basic.

**
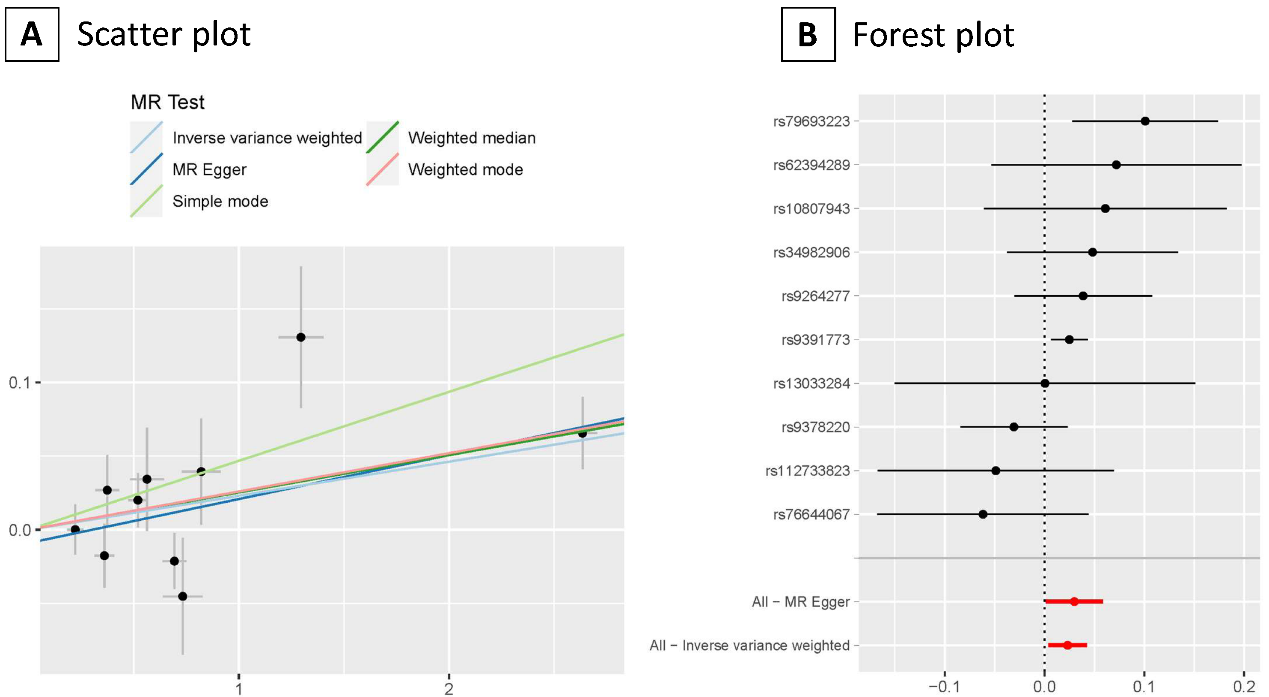
**

Footnotes: AS, ankylosing spondylitis; FGF-basic, basic fibroblast growth factor; MR, mendelian randomization; A, Scatter plot of single-nucleotide polymorphism (SNP) effects on AS vs FGF-basic, with the slope of each line corresponding to estimated MR effect per method. B, Forest plot of individual and combined SNP MR-estimated effect sizes.

**Figure S4.** MR Plots for Relationship of AS with G-CSF.

**
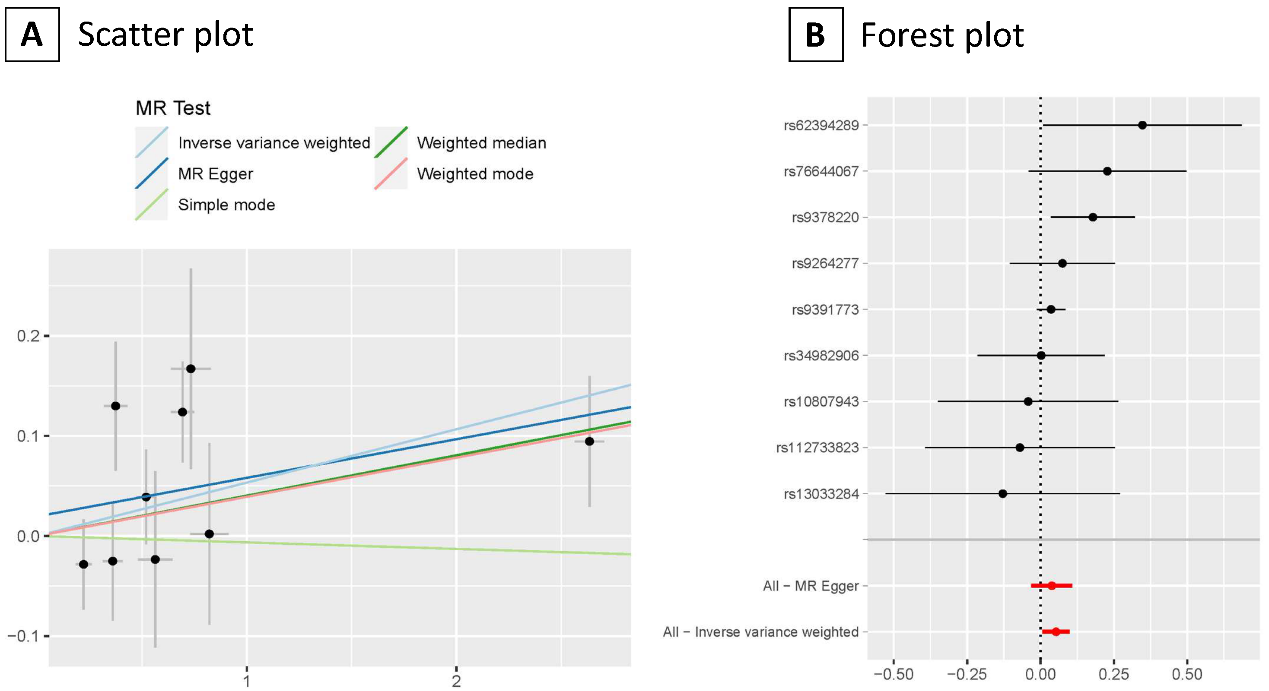
**

Footnotes: AS, ankylosing spondylitis; G-CSF, Granulocyte colony-stimulating factor; MR, mendelian randomization; A, Scatter plot of single-nucleotide polymorphism (SNP) effects on AS vs G-CSF, with the slope of each line corresponding to estimated MR effect per method. B, Forest plot of individual and combined SNP MR-estimated effect sizes.

**Figure S5.** MR Plots for Relationship of AS with MCP-3.

**
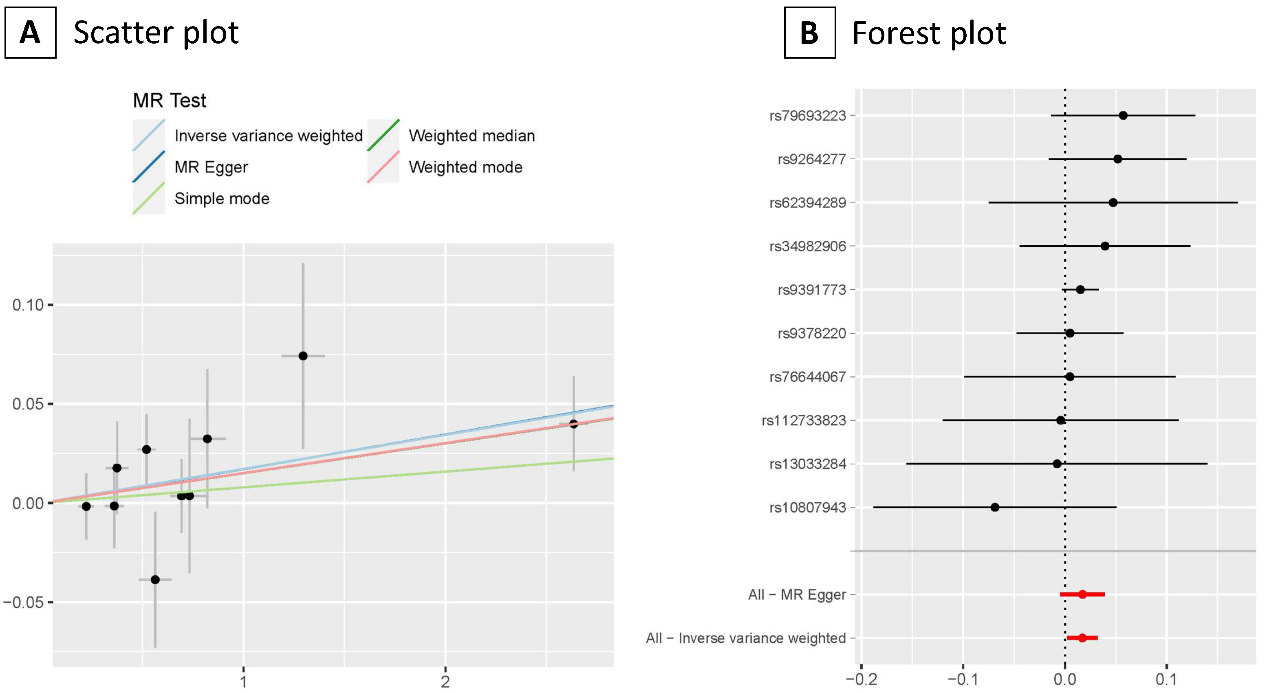
**

Footnotes: AS, ankylosing spondylitis; MCP-3, Monocyte specific chemokine 3 (CCL7); MR, mendelian randomization; A, Scatter plot of single-nucleotide polymorphism (SNP) effects on AS vs MCP-3, with the slope of each line corresponding to estimated MR effect per method. B, Forest plot of individual and combined SNP MR-estimated effect sizes.
